# Evaluation of per- and polyfluoroalkyl substances and organochlorine pesticides in great tit eggs from areas with different anthropogenic pressures in Padova, Italy

**DOI:** 10.1007/s11356-025-37052-5

**Published:** 2025-11-14

**Authors:** Pere Colomer-Vidal, Juan Muñoz-Arnanz, Laura Giovanetti, Ilaria Caliani, Stefania Ancora, Matteo Schiavinato, Flavio Monti, Matteo Beccardi, Marianna Rusconi, Sara Valsecchi, Silvia Casini, Begoña Jiménez

**Affiliations:** 1 Department of Instrumental Analysis and Environmental Chemistry, Institute of Organic Chemistry (IQOG-CSIC), Juan de La Cierva 3, 28006 Madrid, Spain; 2 Department of Physical, Earth and Environmental Sciences, University of Siena, 53100 Siena, Italy; 3 Cluster of Biomolecular Science, Division of Toxicology, Wageningen University and Research, 6708 WE Wageningen, The Netherlands; 4 Institute of Research On Terrestrial Ecosystems (IRET), National Research Council (CNR), Viale Guglielmo Marconi 2, 05010 Porano (TR), Italy; 5 Institute of Avian Research, An Der Vogelwarte 21, 26386 Wilhelmshaven, Germany; 6 Water Research Institute (IRSA), National Research Council (CNR), Via Mulino 19, 20861 Brugherio, MB Italy

**Keywords:** PFAS, OCPs, Great tit, Eggs, PFOS, PFCAs, DDT, Bioindicators

## Abstract

This study investigates the contamination of per- and polyfluoroalkyl substances (PFAS) and organochlorine pesticides (OCPs) in eggs of great tits (*Parus major*) from urban, agricultural, and woodland areas from Italy. PFOS was the most prevalent PFAS, followed by long-chain perfluorocarboxylic acids (PFCAs). Agricultural areas showed the highest PFOS concentrations, while woodland areas exhibited higher concentrations of PFUnDA and PFTrDA. The observed PFCA chain length patterns across different locations highlight the influence of atmospheric chemistry, especially the oxidative breakdown of PFCA precursors, in determining contamination profiles. OCP contamination was dominated by DDT metabolites, especially p,p′-DDE, with higher concentrations in urban areas, reflecting historical pesticide use and the limited soil disturbance in these environments, which prevents the dilution and breakdown of persistent compounds like DDT. Agricultural areas showed elevated PFOS concentrations, likely due to the use of biosolid fertilizers. Principal component analysis revealed distinct contamination patterns across the studied environments, with urban and agricultural areas influenced by local sources, while woodland areas exhibited contamination linked to atmospheric deposition. These results highlight the persistence of both PFAS and OCPs in the environment, underscoring the need for continued monitoring and the utility of great tit eggs as effective bioindicators for assessing the impact of historical and ongoing pollution sources.

**Supplementary Information:**

The online version contains supplementary material available at 10.1007/s11356-025-37052-5.

## Introduction

Birds, particularly their eggs, have long been recognized as effective sentinel species of environmental pollution due to their position in the food chain (Sun et al. 2023). As birds accumulate pollutants from their diet, their eggs reflect the contaminant concentrations present in their habitats (Reindl and Falkowska 2020). This makes bird eggs an ideal medium for monitoring pollutants such as per- and polyfluoroalkyl substances (PFAS) and organochlorine pesticides (OCPs), as their easy collection and the maternal transfer of these compounds provide a reliable means of assessing environmental pollution. Numerous studies, particularly those focused on aquatic birds, have demonstrated the utility of bird eggs in monitoring of PFAS (Colomer-Vidal et al. 2022; Oró-Nolla et al. 2023) and OCPs (Roscales et al. 2017; Zapata et al. 2018).

PFAS are synthetic chemicals recognized for their amphiphilic character, they repel both water (hydrophobic) and oils/fats (lipophobic), and surfactant properties, derived from their highly fluorinated structure and the presence of polar functional groups. These properties made PFAS suitable for a wide range of applications, including fire-fighting foams, textiles, and food packaging (Buck et al. 2011). Their extensive use has resulted in their widespread presence in the environment and human tissues (Giesy and Kannan 2001). Among PFAS, long-chain perfluoroalkyl carboxylic acids (PFCAs) and perfluoroalkyl sulfonic acids (PFSAs) have received significant regulatory attention due to their potential for bioaccumulation and toxicity. Chemicals such as perfluorooctane sulfonate (PFOS) and perfluorooctanoic acid (PFOA) were widely used in the past, but have now been gradually phased out in many areas because of their persistence in the environment and associated health risks (Buck et al. 2011). The early 2000s saw initial voluntary actions by major manufacturers, such as 3M and others in the USA, to phase out C8-based PFAS. Regulatory measures followed at varying speeds, with PFOS being listed in the Stockholm Convention’s annexes in 2009, and PFOA and perfluorooctane sulfonate (PFHxS) added more recently in 2019 and 2022, respectively (UNEP 2022, 2019, 2009). In parallel, initiatives like the Epa’s 2010/2015 PFOA Stewardship Program further accelerated efforts to reduce emissions and eliminate the use of these compounds, reflecting growing regulatory momentum in the USA and the European Union (US EPA 2006). These actions aimed to mitigate the environmental and health impacts of PFAS while allowing limited, controlled uses.

OCPs are characterized by their carbon-chlorine bonds, which contribute to their stability and resistance to environmental degradation. Notable examples include dichlorodiphenyltrichloroethane (DDT) and lindane. DDT, introduced in the 1940 s, revolutionized pest control with its long-lasting effects, significantly reducing the incidence of malaria and other vector-borne diseases (Jones and de Voogt 1999). Lindane was widely used in agriculture and medicine due to its effectiveness against pests and lice (Susman 2001). At the height of their use, OCP production was substantial, with DDT production exceeding 100,000 tons annually (Fernie et al. 2003). Although banned in most parts of the world since the 1970s–1980s (Susman 2001), OCPs still constitute an element of concern because they are easily transported through air and water currents. Their persistence and lipophilicity cause them to bioaccumulate and biomagnify in the environment. Despite regulatory measures, residual concentrations of OCPs persist, impacting wildlife and human health (Jones 2021). Concerns about reproductive disorders, cancer, and endocrine disruption prompted regulatory actions, including the Stockholm Convention on Persistent Organic Pollutants, which sought to eliminate or restrict the most harmful OCPs (Jones 2021). The ongoing presence of OCPs in the environment highlights the need for continuous monitoring and research.

Both PFAS and OCPs are synthetic chemicals known for their environmental persistence, bioaccumulation, and potential health impacts. These pollutants affect avian reproduction by altering parental behaviors, influencing hormonal levels, and impairing reproductive success through reduced hatching rates and thinner eggshells (Blévin et al. 2020; Groffen et al. 2024). Despite their effectiveness in various applications, the environmental and biological consequences of these persistent chemicals raise serious concerns. Although data on terrestrial bird species have been limited, some studies over the past decade have used passerine eggs, particularly those of great tit (*Parus major*), as bioindicator tool of contamination (Van den Steen et al. 2009, 2008), with significant relevance to both PFAS (Groffen et al. 2024, 2019; Lopez-Antia et al. 2021; Morganti et al. 2021) and OCPs (Van den Steen et al. 2009, 2008). Birds, such as great tits, are capital breeders investing a significant amount of energy and lipids into egg production in a short period, which means that the contaminant concentrations in their eggs are expected to reflect the body burden of the female, rather than the contaminants from her diet at the time of laying (Van den Steen et al. 2008). The great tit’s ecological traits make it an excellent model for studying environmental pollution. Its limited migratory tendency and residence in diverse habitats, from agro-forest areas to urban green spaces, allow for the assessment of anthropogenic pressures across different environments. The bird’s small home range, typically around 30 m from the nest, facilitates the monitoring of local contamination without the confounding effect of migration (Van den Steen et al. 2009). Additionally, great tits are cavity-nesters that readily use artificial nest boxes, which simplifies egg collection and monitoring (Lee et al. 2023). Their diet primarily consists of insects, particularly lepidopteran larvae, and they may also consume plant materials, exposing them to anthropogenic substances accumulated in arboreal invertebrates and into vegetables or deposited on the leave surface (Sinkovics et al. 2021). The extensive literature on their ecology, behavior, and reproduction further supports their use as a model species in ecotoxicological studies (Grabowska-Zhang et al. 2012; Hollander et al. 2008; Kvist et al. 2003; Ouyang et al. 2013; Song et al. 2020).

This study aims to investigate the presence of a range of contaminants, with a particular focus on PFAS and OCPs, in great tits living in environments subject to varying concentrations of human impact (wooded, urban, and agricultural areas) around Padua City. By analyzing abandoned and unhatched eggs collected during nests monitoring, we seek to uncover potential environmental pressures and explore the factors behind any observed differences in contaminant concentrations. We hypothesize that urban areas will exhibit higher contamination concentrations due to historical and ongoing human activities, while distinct contamination profiles will emerge across environments, reflecting differences in local and diffuse sources influenced by anthropogenic and ecological factors. Our study offers a unique opportunity to expand on previous research and provide new insights into the contamination of these ecosystems.

## Materials and methods

### Study areas

**Fig. 1:**
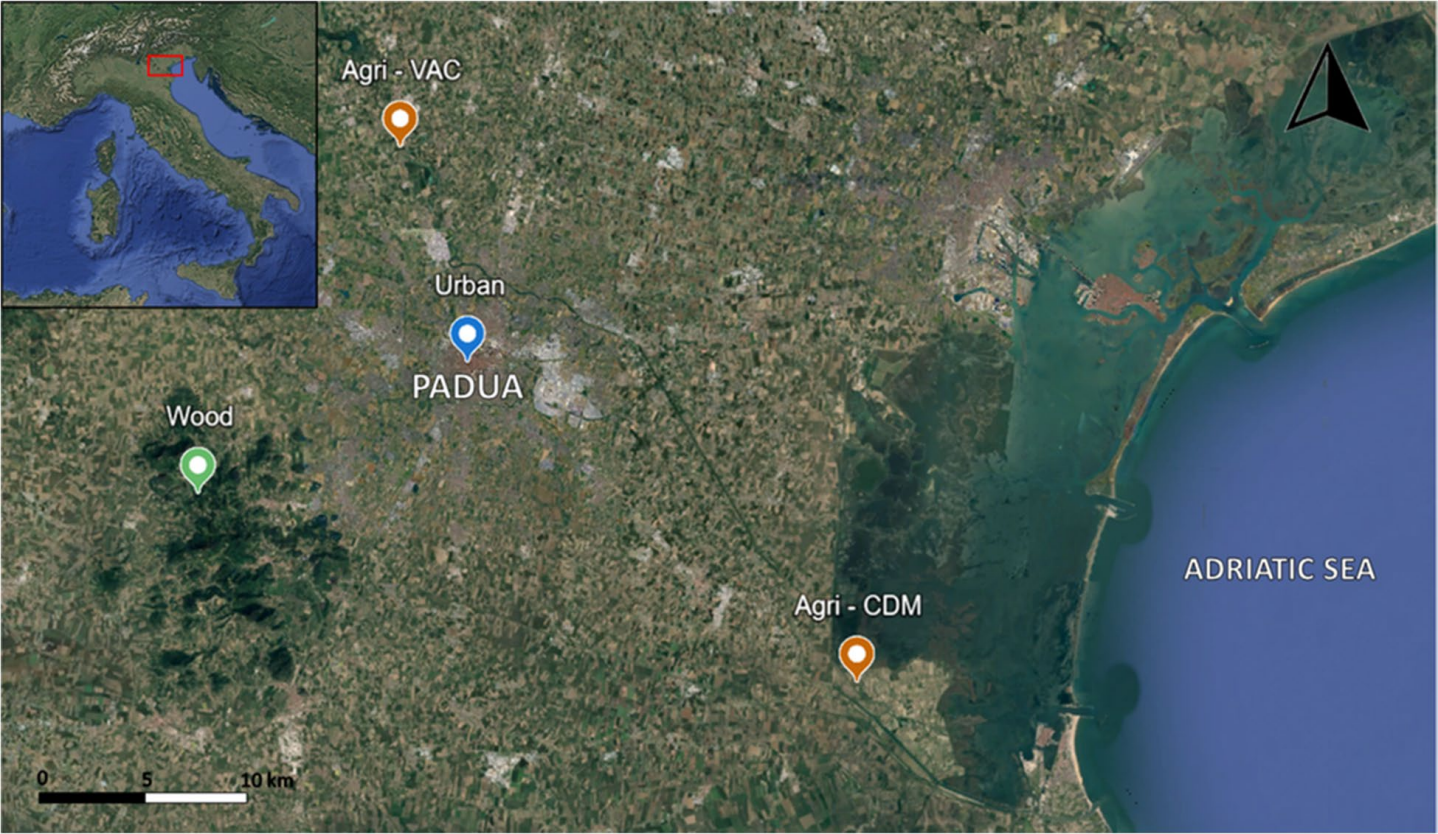
Sampling sites of great tit eggs located in the Veneto Region, Italy: agricultural areas (Agri-CDM and Agri-VAC) in orange, urban site in blue, and woodland area in green

The study areas are located in the Veneto Region, Italy (Fig. 1). Nest boxes were placed both within the city of Padua and its surrounding province, covering various environmental pressures including agricultural, urban, and woodland areas. The study areas are far (> 40 km) from the municipalities of the Veneto Region affected by contamination from the fluorochemical production plant in Trissino, Vicenza (Regione del Veneto 2022). The agricultural areas are represented by Cà di Mezzo Oasis (Agri-CDM; Figure S1), an artificial phytoremediation site that collects wastewater from upstream agricultural lands. It is surrounded by intensive wheat and corn cultivation and located near the Bacchiglione and Brenta rivers, both of which have been contaminated over the years by diffuse pollution from agriculture (fertilizers and pesticides) and by point pollution caused by discharges of urban and industrial wastewater plants (Bertanza et al. 2024; Brambilla et al. 2016; Masiol et al. 2018). Also, Vaccarino (Agri-VAC; Figure S2) is an agricultural area located 15 km from Padua’s center, is bordered by wheat fields, and lies near a busy roadway and the Brenta river. The urban area encompasses three sites (Urban; Figure S3), situated in the center of Padua city (Botanical Garden and Vallisneri) or nearby (S. Antonio Village—Noventa Padovana). The Botanical Garden is the historic Botanical Garden of Padua, located in the northeastern part of the city. Vallisneri is a district in central Padua where nest boxes were installed between the Piovego canal and a major roadway, close to the university complex. Padua is recognized as one of the most air-polluted (PM10, PM2.5, NOx, and O_3_) cities in Europe by the European Environment Agency (EEA 2024a). Villaggio Sant’Antonio is situated in an industrial neighborhood that includes food storage facilities, manufacturing industries, and a factory that produces polyurethane and polyethylene foam panels. The Euganean Hills (Wood; Figure S4) constitute a regional park of volcanic origin located in the southwestern part of Padua province, about 15 km from the city, with elevations ranging from 300 to 600 m above sea level.

### Sample collection

During the 2021 and 2022 breeding seasons, great tit eggs were sampled from nest boxes installed in tree trunks about 3 m above ground. A total of 46 eggs were collected during routine nest inspections after confirming that they were cold, unhatched, or abandoned. Due to the small amount of egg content, we could not use the same egg for both analysis (PFAS and OCPs), but eggs from the same nest were employed for both contaminants. For OCP analysis, 20 eggs were used, while 27 eggs were analyzed for PFAS. The detailed number of eggs per area is provided in Tables 1 and 2. Additional information, including year of collection, area type, location name, nest ID, sample ID and egg status (whole egg, yolk, or embryonated egg) for the great tit eggs used in this study, is available in Table S1.

**Table 1:** PFAS concentrations expressed in ng/g wet weight (mean, median, minimum, and maximum) in samples of great tit eggs from the different studied areas in the Veneto Region, Italy

		PFOA	PFNA	PFDA	PFUnDA	PFDoDA	PFTrDA	PFTeDA	PFOS	PFNS	PFDS	FOSA	∑PFCAs	∑PFSAs	∑PFAS
Agri-CDM	Mean	0.293	0.259	0.901	0.361	0.704	0.272	0.339	7.08	0.0332	0.0320	0.714	2.79	7.10	9.99
*n* = 7	Median	0.302	0.166	0.822	0.293	0.649	0.103	0.189	6.30	0.0285	0.0320	0.714	2.44	6.32	8.93
	Min	0.084	LOD	0.0292	LOD	0.119	LOD	LOD	2.58	LOD	LOD	LOD	0.794	2.58	3.37
	Max	0.463	0.562	1.72	0.731	1.76	0.629	0.872	11.9	0.0514	0.0320	0.714	6.45	11.9	18.3
Agri-VAC	Mean	0.322	0.598	0.901	0.343	0.986	0.990	0.0365	4.82	LOD	LOD	LOD	3.66	4.82	8.48
*n* = 4	Median	0.317	0.612	0.910	0.275	0.877	0.990	0.0365	5.10	LOD	LOD	LOD	3.19	5.10	7.82
	Min	0.219	0.453	0.652	0.0180	0.685	LOD	LOD	3.75	LOD	LOD	LOD	2.64	3.75	7.41
	Max	0.439	0.716	1.13	0.804	1.50	1.204	0.0413	5.31	LOD	LOD	LOD	5.63	5.31	10.9
Urban	Mean	0.284	0.359	1.09	0.428	2.01	0.615	0.961	4.68	0.0238	0.0425	LOD	5.37	4.69	10.1
*n* = 9	Median	0.240	0.279	0.873	0.314	1.75	0.462	0.812	3.65	0.0238	0.0425	LOD	4.48	3.65	9.37
	Min	0.169	0.132	0.442	LOD	0.676	LOD	LOD	1.95	LOD	LOD	LOD	1.92	1.95	3.88
	Max	0.534	0.899	2.35	1.38	3.95	1.36	2.50	10.0	0.0238	0.0425	LOD	13.0	10.1	23.0
Woodland	Mean	0.228	0.773	1.16	1.06	1.48	1.39	0.808	2.43	LOD	LOD	LOD	5.64	2.43	8.07
*n* = 6	Median	0.224	0.418	0.743	0.529	1.13	1.02	0.501	2.23	LOD	LOD	LOD	4.15	2.23	6.51
	Min	0.127	LOD	0.457	LOD	LOD	LOD	LOD	0.362	LOD	LOD	LOD	0.127	0.362	0.489
	Max	0.342	2.28	3.01	3.13	3.13	3.20	1.88	5.52	LOD	LOD	LOD	16.9	5.52	22.4
Overall	Mean	0.279	0.460	1.02	0.537	1.37	0.814	0.651	4.83	0.0313	0.0372	0.714	4.47	4.84	9.34
*n* = 26	Median	0.272	0.346	0.822	0.402	0.982	0.645	0.334	4.75	0.0267	0.0372	0.714	3.18	4.77	7.69
	Min	0.084	LOD	0.0292	LOD	LOD	LOD	LOD	0.362	LOD	LOD	LOD	0.127	0.362	0.489
	Max	0.534	2.28	3.01	3.13	3.95	3.20	2.50	11.9	0.0514	0.0425	0.714	16.9	11.9	23.0

**Table 2:** OCP concentrations expressed in ng/g lipid weight (lw) (mean, median, minimum, and maximum) in samples of great tit eggs from the different studied areas in the Veneto Region, Italy

		PeCB	HxCB	α- HCH	γ- HCH	β- HCH	∑HCHs	α- endosulfan	β- endosulfan	∑endosulfan	op′-DDE	pp′-DDE	op′-DDD	op′-DDT	pp′-DDD	pp′-DDT	∑DDTs	∑OCPs
Agri-CDM	Mean	0.89	16.7	0.831	0.981	2.15	3.76	0.326	0.321	0.390	0.255	4110	0.123	0.692	7.52	41.6	4160	4180
*n* = 5	Median	0.796	14.2	0.614	0.981	1.29	4.13	0.336	0.321	0.330	0.222	3170	0.113	0.671	1.55	10.5	3180	3210
	Min	0.517	11.1	0.195	LOD	0.565	1.49	0.141	LOD	0.141	0.029	2570	0.0212	0.196	0.712	7.98	2580	2610
	Max	1.38	26.1	1.84	1.05	4.62	6.11	0.536	0.321	0.858	0.601	8370	0.286	1.42	31.3	167	8570	8590
Agri-VAC	Mean	0.853	5.54	0.868	2.85	0.472	4.19	0.265	LOD	0.265	0.351	522	0.109	0.353	3.09	9.76	536	548
*n* = 2	Median	0.855	5.54	0.868	2.85	0.472	4.19	0.265	LOD	0.265	0.351	522	0.109	0.353	3.09	9.76	536	548
	Min	0.652	5.03	0.788	1.64	0.315	2.74	0.241	LOD	0.241	0.349	493	0.071	0.324	2.91	9.05	505	515
	Max	1.06	6.04	0.949	4.05	0.628	5.63	0.296	LOD	0.295	0.354	552	0.147	0.382	3.27	10.5	567	581
Urban	Mean	1.26	11.9	0.813	0.865	0.809	2.25	0.235	0.167	0.355	0.214	1790	0.186	1.25	3.98	32.0	1830	1840
*n* = 7	Median	0.955	12.4	0.826	0.733	0.900	2.25	0.201	0.188	0.342	0.167	1800	0.119	1.19	3.23	32.0	1830	1850
	Min	0.844	5.62	LOD	0.471	0.0910	0.573	0.138	LOD	0.168	LOD	1590	LOD	0.458	2.51	20.6	1610	1620
	Max	2.21	17.6	1.12	1.65	1.68	3.99	0.414	0.204	0.601	0.476	2050	0.353	2.50	7.25	39.3	2090	2110
Woodland	Mean	1.11	19.2	0.879	0.856	1.71	3.44	0.266	0.182	0.303	0.234	609	0.112	0.569	1.64	15.1	627	651
*n* = 6	Median	1.14	20.4	0.762	0.612	1.21	2.84	0.186	0.182	0.292	0.233	628	0.112	0.557	1.60	11.5	642	670
	Min	0.745	11.2	0.458	0.0517	0.805	1.37	0.112	LOD	LOD	0.098	309	0.0664	0.356	0.33	5.84	316	341
	Max	1.41	25.7	1.88	2.85	4.36	6.05	0.467	0.182	0.467	0.367	910	0.152	0.863	3.59	31.9	947	965
Overall	Mean	1.08	14.6	0.846	1.09	1.38	3.18	0.273	0.192	0.341	0.249	1890	0.127	0.817	4.08	27.1	1920	1940
*n* = 20	Median	1.08	14.1	0.807	0.875	1.12	2.86	0.247	0.188	0.318	LOD	1680	0.116	0.616	2.96	17.7	1710	1730
	Min	0.512	5.03	LOD	LOD	0.0910	0.574	0.118	LOD	LOD	0.029	309	LOD	0.19	0.330	5.84	316	341
	Max	2.21	26.0	1.88	4.05	4.62	6.11	0.536	0.321	0.858	0.601	8370	0.353	2.50	31.3	167	8570	8590

### Sample preparation and extraction

The eggs were stored at − 80 °C until processing. Once thawed, they were homogenized and lyophilized to remove their aqueous content. Detail of the analytical considerations and extraction procedures is described in the supplementary material (Text S1).

#### PFAS

For PFAS analysis, following Mazzoni et al. (2016), about 0.1–0.2 g of the dry egg content was placed in a centrifuge tube, spiked with a stable isotope labeled solution used as internal standard mixture solution, and extracted with a water-acetonitrile mixture (10:90, v/v) and formic acid. The sample underwent ultrasonication, centrifugation, and was repeated twice. The combined supernatants were treated with MgSO_4_ and NaCl, stored overnight at − 4 °C, and reduced to 1 mL. Phospholipids were removed using HybridSPE® Phospholipid Ultra cartridges (Sigma-Aldrich, St. Louis, MO, USA) after acidifying the extract. Details on the analyte names and abbreviations are reported in Table S2.

#### OCPs

For OCP analysis, approximately 0.2 g of freeze-dried egg sample was homogenized with 1.5 g of anhydrous sodium sulfate to remove residual moisture and fortified with isotopically labeled standards of ^13^C_6_-PeCB, ^13^C_6_-HxCB, ^13^C_6_-α-HCH, ^13^C_6_-ɣ-HCH, ^13^C_10_-p,p-DDE, ^13^C_10_-o,p′-DDT, ^13^C_10_- p,p′-DDT, ^13^C_9_-α-endosulfan, and ^13^C_9_-β-endosulfan. The mixture was placed into a glass cell, soaked with 10 mL of cyclohexane:acetone (3:1, v/v), and subjected to an ultrasound assisted extraction by means of an ultrasonic bath (Ultrasonic Cleaner 2.6 L/3000683, J.P. Selecta) at room temperature for 15 min, repeated three times. The combined extracts (≈30 mL) were concentrated to 1 mL using a TurboVap® system under nitrogen at 40°C. For purification, gel permeation chromatography (GPC) was employed to separate the lipid content. The eluate fraction from GPC (110mL) containing the target analytes was further concentrated to 1 mL and further cleaned up via adsorption chromatography on a silica column modified with sulfuric acid at 44% (w/w), followed by elution with n-hexane:dichloromethane (9:1, v/v). The purified extracts (volume) were concentrated to almost dryness under nitrogen and reconstituted with a specific amount of isotopically labeled injection standards of PCBs, prior to instrumental analysis. The lipid content was determined gravimetrically from the corresponding eluate fraction from GPC (volume) weighing the residue after solvent evaporation. Comprehensive information about this method can be found in the SI.

### Instrumental analysis

#### PFAS

PFAS in the final extract were determined by liquid chromatography tandem mass spectrometry (UHPLC-MS/MS) coupled to turbulent flow chromatography (TFC) for the online purification of the extracts (Mazzoni et al. 2016). Legacy and novel PFAS were determined using a modified Thermo EQuan system, which consists of a CTC PAL autosampler equipped with four six-way VICI valves, two Thermo Scientific Accela LC pumps (600 and 1200) equipped with serially connected TFC (Thermo Fluoro XL, 50 × 0.5 mm and Thermo CycloneTM,50 × 0.5 mm) and analytical (Waters Acquity UPLC BEH C18, 1.7 µm 2.1 × 50 mm) columns. The TFC system was connected to a triple quadrupole mass spectrometer (Thermo Scientific TSQ Quantum Access MAX) with a heated-electrospray ionization (HESI-II) probe operated under negative ion and selected reaction monitoring (SRM) transitions (see Table S2 for parent and product ions). Fifty microliters of extract was injected and loaded into the TFC columns using 1% HCOOH in water as eluent at 2000 mL/min. Analytes are then eluted by a MeOH plug and analyzed by the following gradient method at 0.3 mL/min using LC–MS grade methanol (mobile phase B) and 2 mM ammonium acetate/5% MEOH (mobile phase A): the initial solvent composition was 95% A and 5% B. Initial condition was held for 1 min, increased to 70% B by 4 min, 100% B by 9 min, hold until 12.5 min, then returned to initial conditions by 1 min and equilibrated for 10 min before the next run. In order to delay the interfering background peaks of PFAS, which can be present in solvents or released by the analytical system, a trap column (Thermo Hypersil GOLD 1.9 µm, 50 2.1 mm) was placed between the analytical pump and the injection valve.

#### OCPs

Identification and quantification of organochlorine pesticides (PeCB, HCB, p,p′-DDT, o,p′-DDT, p,p′-DDE, o,p′-DDE, p,p′-DDD, o,p′-DDD, α-HCH, β-HCH, γ-HCH, α-endosulfan, and β-endosulfan) were carried out using GC coupled to a low-resolution tandem mass spectrometer with a triple quadrupole analyzer (GC-QqQ-MS/MS) (Muñoz-Arnanz et al. 2024). Helium was used as carrier gas. The gas chromatograph used was a 7890B (Agilent, Palo Alto, CA, USA) equipped with a programmable temperature vaporization injector (PTV) operating in splitless mode and a DB35-ms column (30 m × 0.25mm × 0.25 µm, Agilent J&W, USA). The mass spectrometer was a 7010B (Agilent, Palo Alto, CA, USA) equipped with a high-efficiency 70eV electron impact ionization source. A source temperature of 260 °C and quadrupole temperatures of 150 °C were used. A volume of 1 μL was injected, and the multiple reaction monitoring (MRM) mode was employed, specifically monitoring two transitions for each analyte. Further information can be found in the SI.

### Quality assurance

#### PFAS

Quantification was performed by the isotopic dilution method, and calibration curves were acquired in each analytical run. Calibration curve standards (0–100 µg/L) were daily prepared diluting certified analytical mixed standard solutions with acetonitrile, which were acidified to pH 3 and spiked with IS by adding 50 µL of concentrated formic acid and 100 µL of the IS solution (40 µg/L) to 0.9 mL of mixed standard solution.

Procedural blanks were run for every extraction batch; their PFAS concentrations were always below respective limits of detection (LODs). LODs and limits of quantification (LOQs) were estimated according to the International Organisation for Standardisation (ISO 6107‐2:2006, 2006) as, respectively, threefold and tenfold the standard deviation of an extract of biological tissue fortified at 1 μg/L. The LODs and LOQs values were 0.01 to 0.05 and 0.02 to 0.15 ng/g dw, respectively.

#### OCPs

A blank sample was included every five egg samples to ensure quality control throughout the entire analytical process. The identification and quantification of target analytes were confirmed using the following criteria: (a) matching GC retention times within ± 0.1 min of those of the standard compounds, (b) monitored ion or MRM ratios within ± 15% of the expected values, and (c) for the case of GC–MS/MS, instrumental LOQs (iLOQs) were calculated as 10 times the standard deviation (SD) of six replicate injections of the lowest point in the calibration curve. All analyte concentrations were adjusted for recovery rates and blank values when applicable. Calibration curves were verified on a daily basis. Detailed information on QA/QC, including recovery and LOD data, can be found in the SI.

### Statistical analysis

Data were expressed in ng/g ww (wet weight) for PFAS, as they primarily bind to proteins, and ng/g lw (lipid weight) for OCPs due to their lipophilic nature. Statistical analyses were performed using R-4.3.1 software (https://www.r-project.org/). The normality and heteroscedasticity of target compound data (including both raw and log-transformed data) were assessed using the Shapiro–Wilk and Levene tests, respectively. As the data (both raw and log-transformed) did not follow a normal distribution (Shapiro–Wilk and Levene tests, *p* < 0.05), non-parametric tests were used.

Data were presented log transformed in graphical format considering all locations, while statistical analysis was performed, also log transformed, considering the type of area (agricultural, urban, and woodland). The Wilcoxon test was used to determine whether there were any significant differences in PFAS and OCP concentrations between 2021 and 2022. To determine the influence of the study area on PFAS and OCP concentrations in the eggs of the great tit, Kruskal–Wallis test (*p* < 0.05) and Dunn’s test using the Benjamini-Hochberg (*p* < 0.05) method were conducted. A principal component analysis (PCA) was performed on the raw data to examine the association patterns among PFAS and OCPs across the study area. The usefulness of the PCA was assessed using the Kaiser–Meyer–Olkin (KMO) measure of sampling adequacy. KMO ranges from 0 to 1 and should be well above 0.5 for PCA to be useful when variables are sufficiently interdependent. Values below the limit of detection (LOD) were considered half of the LOD (Van den Steen et al. 2009, 2008; Groffen et al. 2019). Additionally, a post hoc statistical power analysis was performed for the Kruskal–Wallis tests assessing differences in PFAS and OCP concentrations across area types. Observed effect sizes (Epsilon-squared, *η*^2^) were converted to Cohen’s *f* to estimate statistical power. All eggs were included in the statistical analysis (Table S1).

## Results

No significant differences were found in PFAS and OCP concentrations between 2021 and 2022 (see Tables S3 and S4). Given the lack of significant variation between the 2 years, the data were combined for a more streamlined analysis. Post hoc power analysis showed that statistical power ranged from very low (< 0.10) for compounds with minimal effect sizes to ≥ 0.90 for those with large effect sizes and statistically significant differences (Table S5). The mean, median, minimum, and maximum concentrations of PFAS and OCPs are shown in Tables 1 and 2, respectively.

### PFAS

**Fig. 2:**
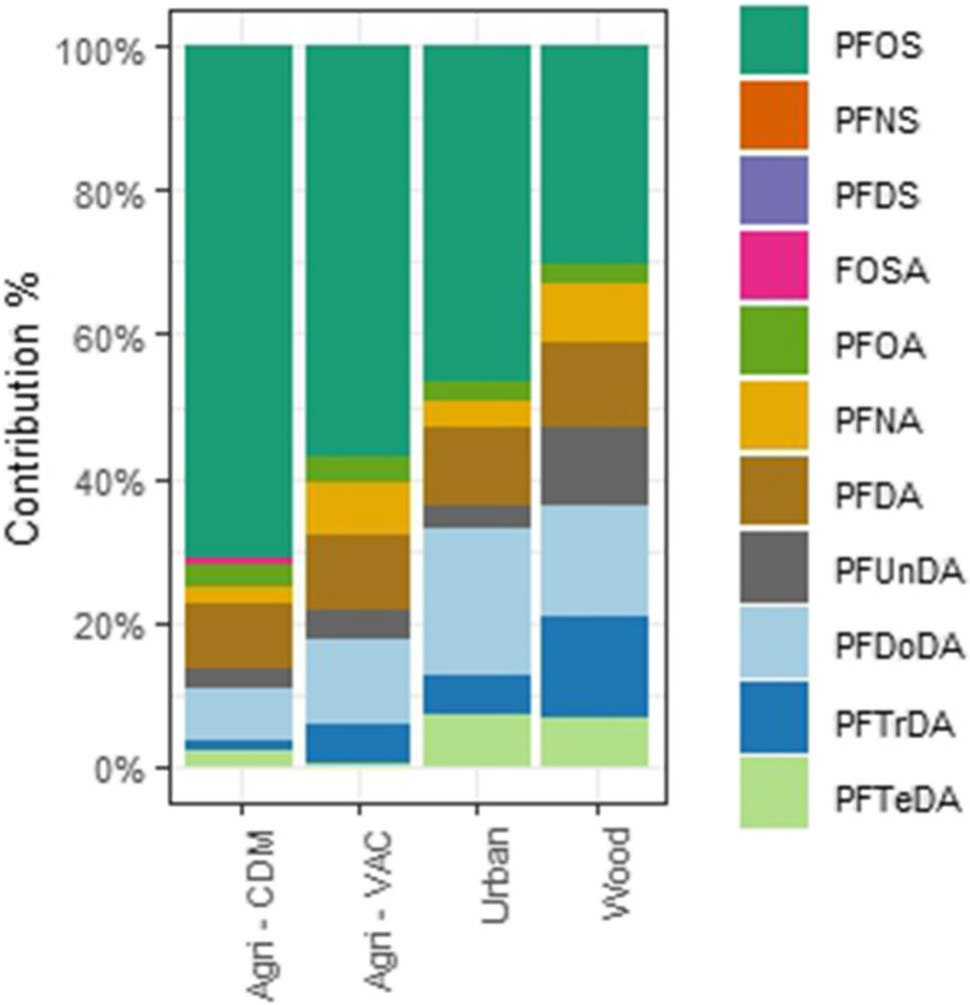
Contribution of PFAS in great tit eggs from the different studied areas in the Veneto Region, Italy

Among the different PFAS detected, PFOS was the most frequent, followed by long-chain PFCAs (Fig. 2). However, the contribution of PFOS varied across locations. Agricultural areas exhibited the highest PFOS contribution (55–70%), while wood area showed the lowest (35%). In urban area, the PFOS contribution was consistently around 50%. PFDoDA and PFDA were the predominant PFCAs, with relatively consistent contributions across all locations. Interestingly, the woodland area showed a higher contribution of PFUnDA and PFTrDA compared to the other areas.

**Fig. 3:**
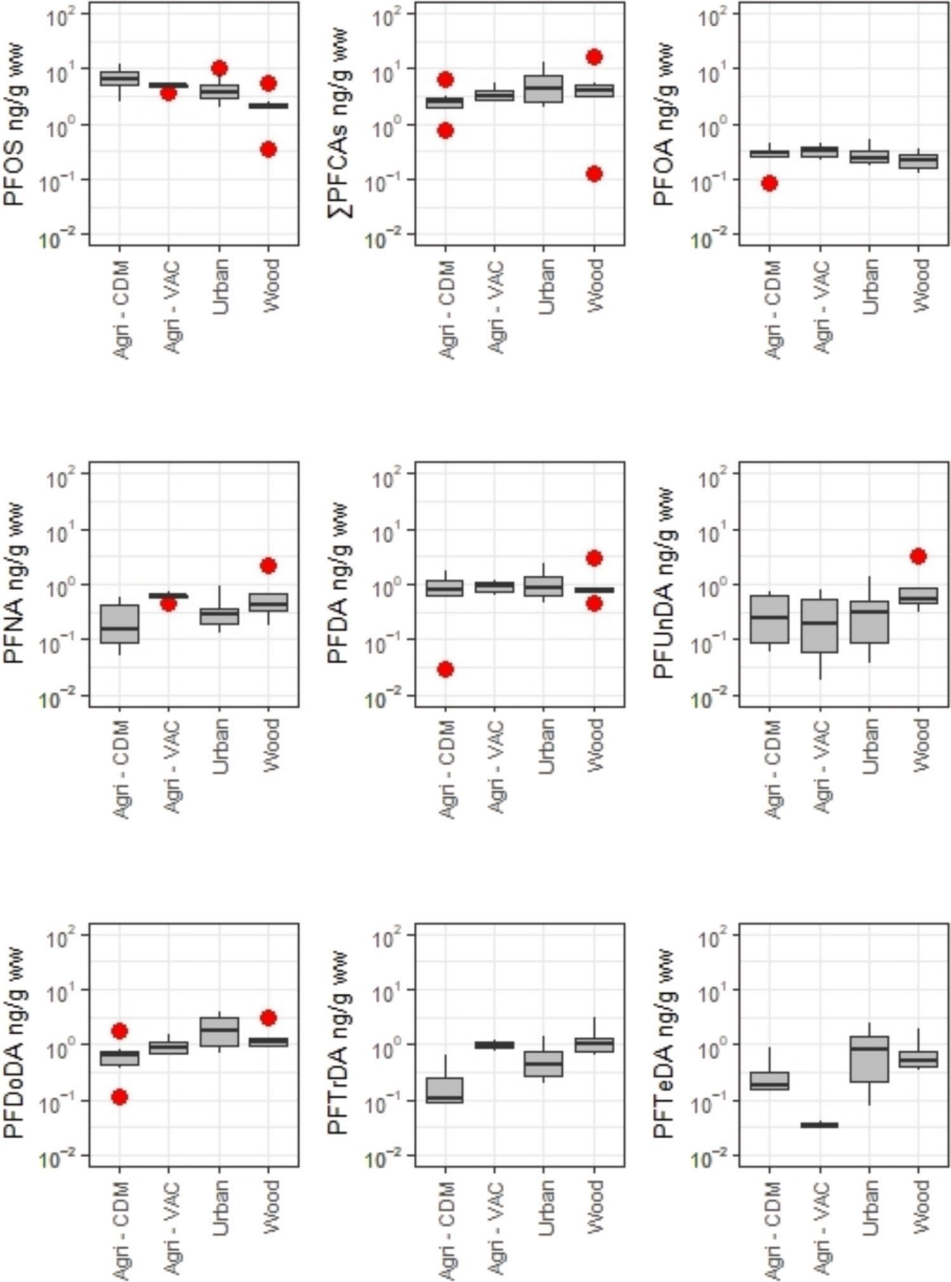
PFOS and PFCA concentrations (ng/g ww in log scale) in great tit eggs from the different studied areas in the Veneto Region, Italy. Red dots represent outliers

∑PFAS concentrations ranged from 0.489 to 23.0 ng/g ww, with a median of 7.69 ng/g ww. As the dominant PFAS, PFOS concentrations ranged from 0.361 to 11.9 ng/g ww, while PFDoDA and PFDA were the predominant PFCAs, with concentrations ranging from 0.119 to 3.95 ng/g ww and 0.0292 to 3.01 ng/g ww, respectively. The highest median PFOS concentrations were found in agricultural areas (Agri-CDM: 6.30 ng/g ww and Agri-VAC: 5.10 ng/g ww), followed by urban and woodland areas (3.65 and 2.23 ng/g ww, respectively) (Fig. 3). ∑PFCAs showed the highest median values in urban areas, largely due to PFDA, PFDoDA, and PFTeDA. However, PFOA and PFNA exhibited their highest median values in Agri-VAC, while PFUnDA and PFTrDA reached their peaks in woodland area (Fig. 3). No significant differences were observed between locations, except for PFOS and PFDoDA (*p* < 0.05; Table S6). The differences in PFOS concentrations were exclusively attributed to the higher median concentrations detected in Agri-CDM (6.30 ng/g ww) compared to woodland area (2.23 ng/g ww). In contrast, PFDoDA differences were attributed to the higher concentrations detected in urban and woodland areas (1.75 and 1.13 ng/g ww) compared to Agri-CDM (0.649 ng/g ww) (*p* < 0.05; Table S7). Agri-CDM showed significantly lower concentrations of PFNA compared to Agri-VAC and of PFTrDA compared to the woodland area, while Agri-VAC had significantly lower concentrations of PFTeDA compared to both urban and woodland areas (*p* < 0.05; Table S7).

**Fig. 4:**
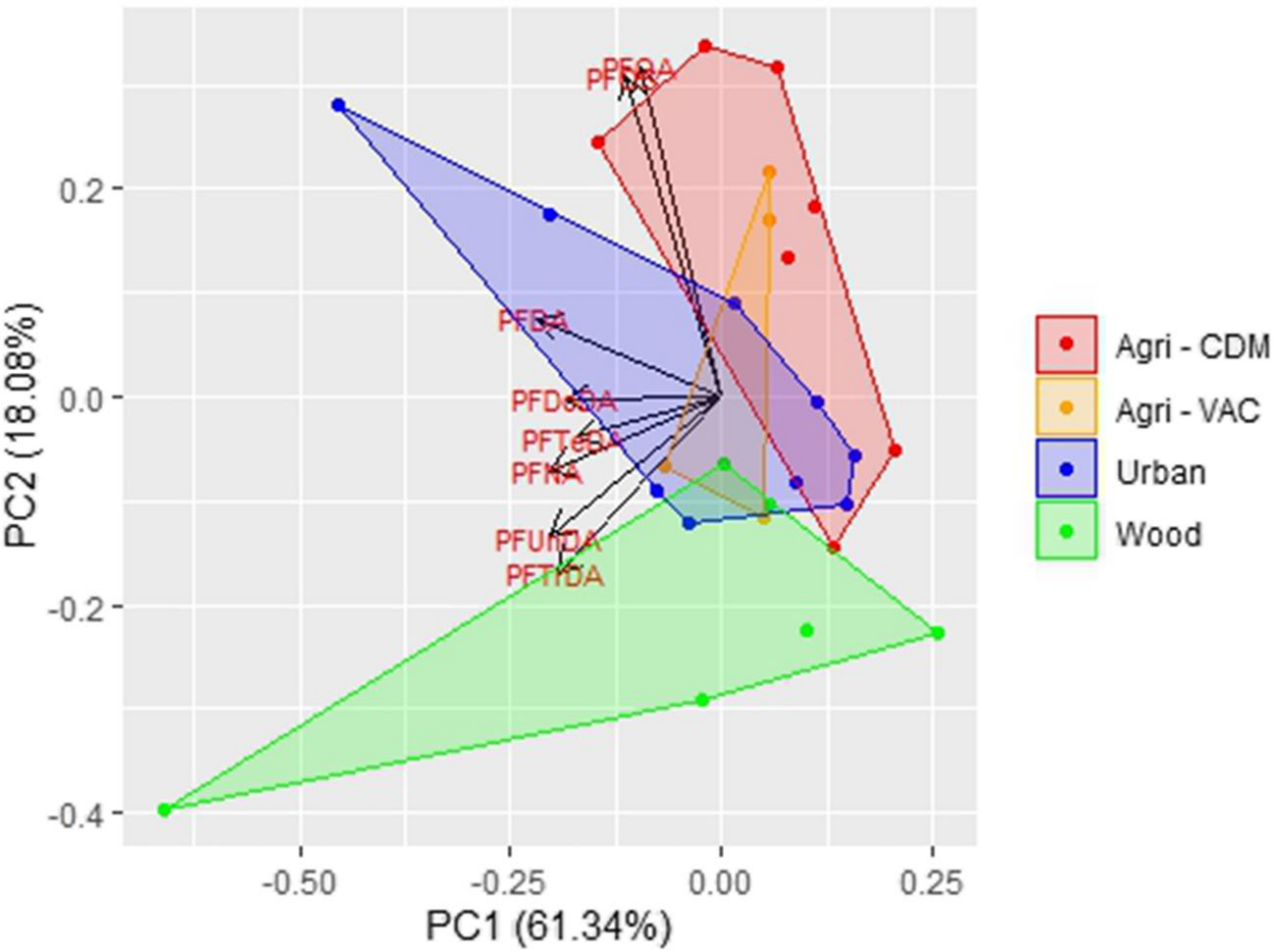
Score plot of PFAS (ng/g ww) in great tit eggs from the different studied areas in the Veneto Region, Italy

In the score plot of PFAS compounds across various areas, the KMO measure of sampling adequacy (MSA) of 0.68 indicates moderate suitability for PCA, effectively summarizing the variability in the data across the studied areas (Fig. 4, Table S8). The first two principal components, PC1 and PC2, explain a combined 79.42% of the total variability (61.34% and 18.08%, respectively). The studied areas exhibited a clear separation between agricultural, urban, and woodland sites, highlighting differences in the analyzed variables. Specifically, agricultural areas (both Agri-CDM and VAC) were more influenced by PFOS and PFOA, while woodland and urban areas were strongly associated with long chain PFCAs (nC > 8). Woodland areas showed a strong correlation with odd-numbered PFCAs like PFUnDA and PFTrDA, while urban areas were more influenced by even-numbered PFCAs such as PFDA, PFDoDA, and PFTeDA.

### OCPs

**Fig. 5:**
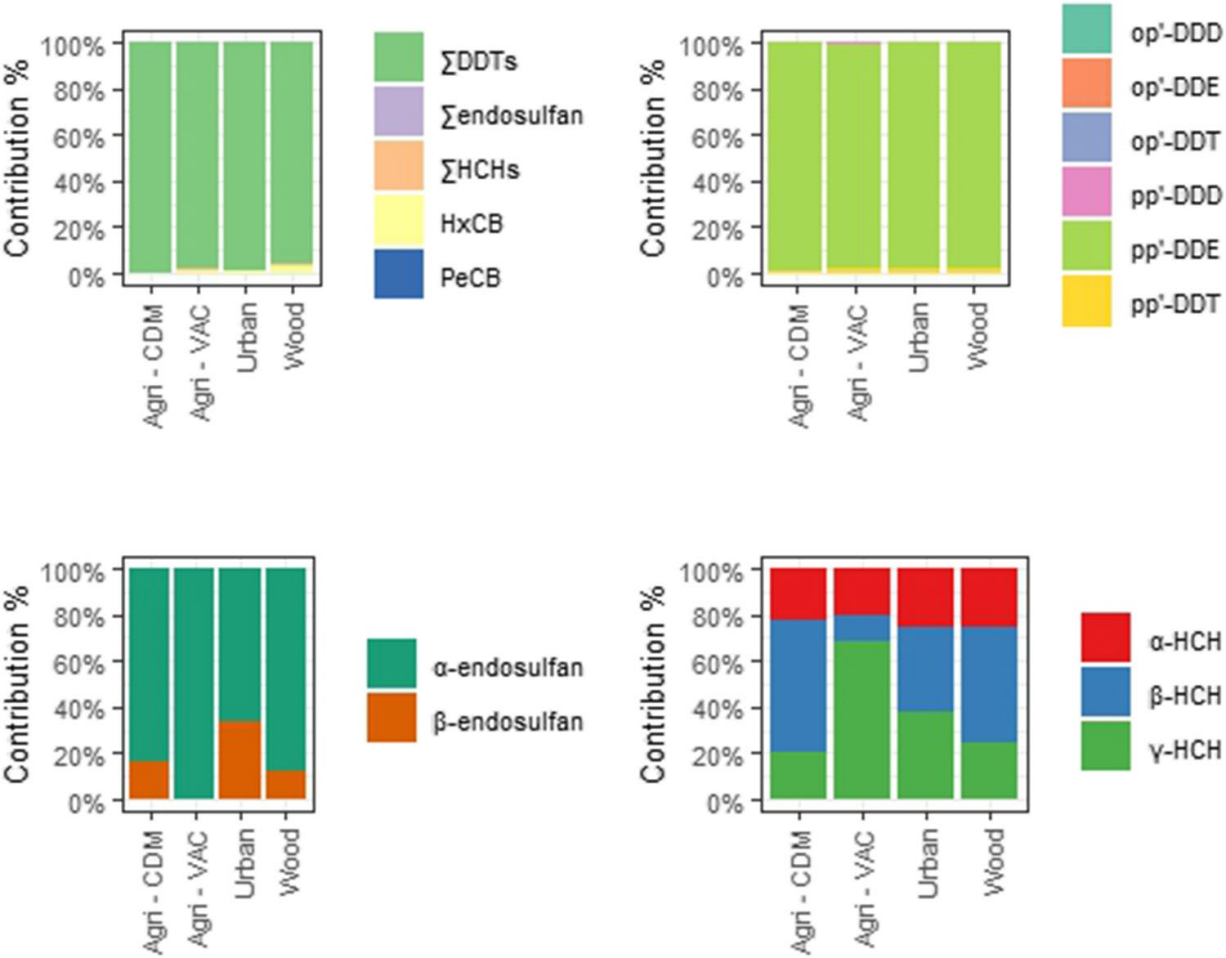
Contribution of OCP families (top left), DDTs (top right), endosulfan (bottom left), and HCHs (bottom right) in great tit eggs from the different studied areas in the Veneto Region, Italy

**Fig. 6:**
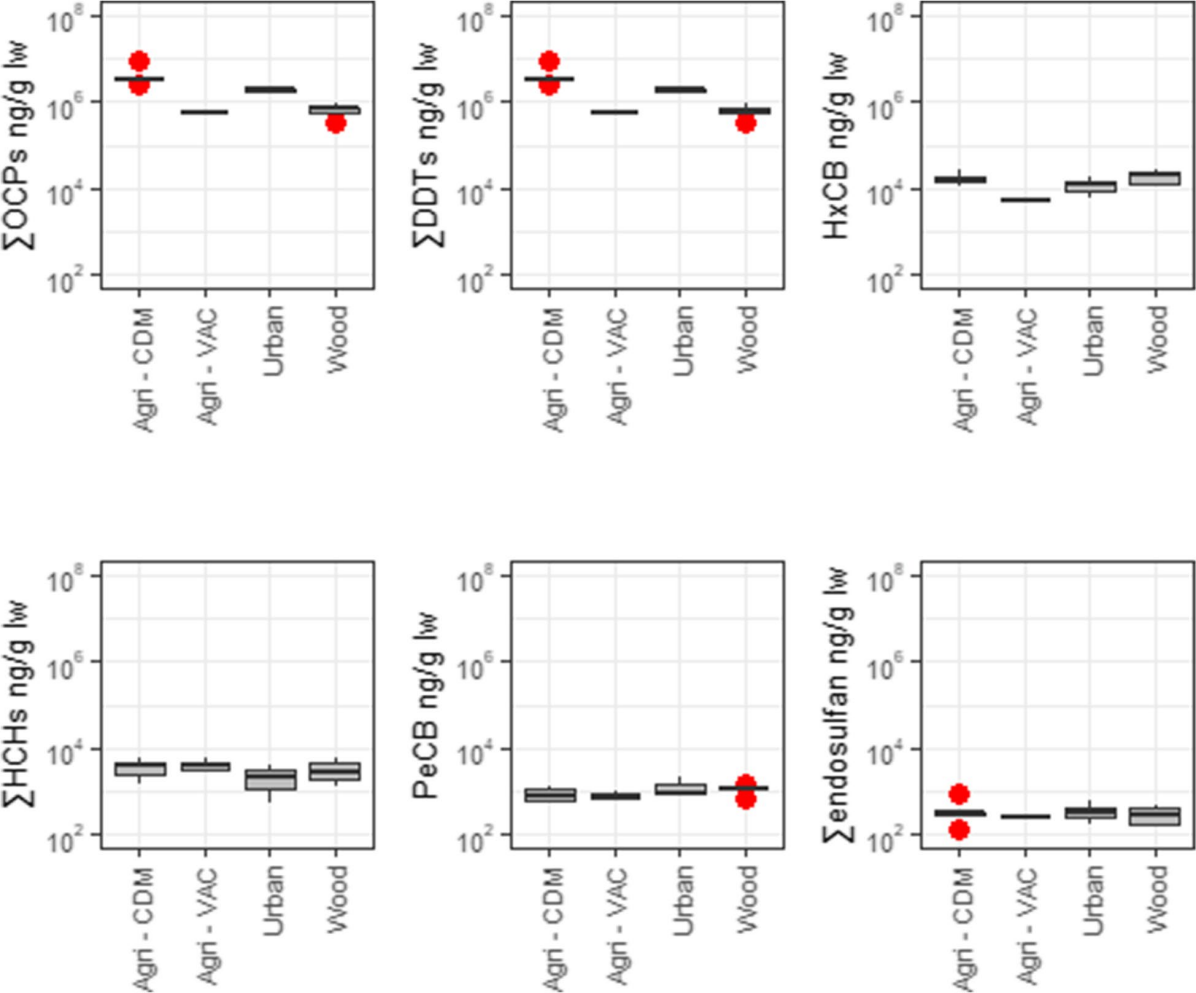
∑OCPs levels (ng/g lw in log scale) and main families in great tit eggs from the different studied areas in the Veneto Region, Italy. Red dots represent outliers

∑OCP concentrations ranged from 341 to 8590 ng/g lw, with a median of 1730 ng/g lw. In all locations, the ∑OCP profile was consistent, dominated by ∑DDTs (1710 ng/g lw), followed by HCB, ∑HCHs, PeCB, and ∑endosulfan (14.1, 3.45, 1.08, and 0.305 ng/g lw, respectively; Fig. 5). Agri-CDM showed the highest median level of ∑OCPs, followed by urban, woodland areas, and Agri-VAC (Fig. 6). Significant differences in ∑OCP concentrations were observed between locations (*p* < 0.05; Table S9), mainly due to the higher concentrations detected in Agri-CDM (3210 ng/g lw) compared to woodland area and Agri-VAC (670 and 548 ng/g lw, respectively), and between urban (1850 ng/g lw) and woodland area (*p* < 0.05; Table S10).

The primary contributor to the ∑DDTs profile (1710 ng/g lw; range: 316–8570 ng/g lw) in all locations was p,p′-DDE (1,680 ng/g lw), followed by p,p′-DDT, p,p′-DDD, o,p′-DDT, o,p′-DDE, and o,p′-DDD (17.6, 2.96, 0.616, 0.223, and 0.116 ng/g lw, respectively). Among the DDT isomers, only p,p′-DDE and p,p′-DDT exhibited significant differences between locations (*p* < 0.05; Table S9). Pairwise comparisons showed that p,p′-DDE, the predominant compound of both ∑DDTs and ∑OCPs, was significantly higher in Agri-CDM (3170 ng/g lw) compared to Agri-VAC and woodland area (552 and 628 ng/g lw, respectively), and between urban (1800 ng/g lw) and woodland area. Additionally, p,p′-DDT median concentrations were significantly higher in urban areas (32.0 ng/g lw) compared to woodland area (11.5 ng/g lw). Also, urban area showed a significantly higher concentration than woodland area for p,p′-DDD and Agri-VAC for o,p′-DDT (*p* < 0.05; Table S10). The R_o,p′/p,p′_ ratio ([o,p′-DDT]/[p,p′-DDT]) was used to differentiate between dicofol and technical DDT usage, with a ratio greater than 0.2 indicating dicofol-type contamination. The R_p,p′/p,p′_ ratio ([p,p′-DDT]/[p,p′-DDE] + [p,p′-DDD]) was employed to distinguish between fresh and weathered residues, with a value of 0.5 serving as the benchmark for differentiating legacy from recent inputs (Muñoz-Arnanz and Jiménez 2011). According to the R_o,p′_/_p,p′_ ratio, technical DDT dominated in all samples, while the R_p,p′/p,p′_ ratio indicated that all detected DDTs were of legacy origin (Table 3).

**Table 3:** Ratios of HCH and DDT isomers

		α-HCH/γ-HCH	β-HCH/(α-HCH + γ-HCH)	p,p′-DDT/(p,p′-DDE + p,p′-DDD)	o,p′-DDT/p,p′-DDT
Agri-CDM	Mean	0.990	2.47	0.00670	0.0410
*n* = 5	Median	0.783	1.93	0.00320	0.0337
	Min	0.643	0.292	0.00250	0.00850
	Max	1.749	6.62	0.0198	0.0841
Agri-VAC	Mean	0.357	0.128	0.0186	0.0362
*n* = 2	Median	0.357	0.128	0.0186	0.0362
	Min	0.234	0.128	0.0183	0.0358
	Max	0.480	0.130	0.0188	0.0365
Urban	Mean	0.943	0.503	0.0178	0.0392
*n* = 7	Median	0.839	0.541	0.0180	0.0400
	Min	0.396	0.192	0.0129	0.0143
	Max	1.506	0.730	0.0226	0.0666
Woodland	Mean	3.583	1.40	0.0230	0.0502
*n* = 6	Median	1.78	1.41	0.0209	0.0367
	Min	0.530	0.279	0.0137	0.0255
	Max	10.7	3.30	0.0349	0.0945
Overall	Mean	1.82	1.23	0.0166	0.0427
*n* = 20	Median	0.839	0.607	0.0181	0.0364
	Min	0.234	0.126	0.00250	0.00850
	Max	10.7	6.62	0.0349	0.0945

HCB concentrations ranged from 5.03 to 26.0 ng/g lw, with a median concentration of 14.1 ng/g lw. While no significant differences were observed between locations overall (*p* > 0.05; Table S9), pairwise comparisons revealed that Agri-CDM (20.4 ng/g lw) and the woodland area (14.2 ng/g lw) had significantly higher concentrations compared to Agri-VAC (5.54 ng/g lw) (*p* < 0.05; Table S10). PeCB concentrations ranged from 0.514 to 2.21 ng/g lw, with a median concentration of 1.08 ng/g lw. No significant differences were detected between locations or in pairwise comparisons (*p* > 0.05; Tables S8 and S9). Among the locations, the woodland area exhibited the highest median concentrations (1.14 ng/g lw), while Agri-VAC had the lowest (0.79 ng/g lw).

The concentrations of ∑HCHs ranged from 1.16 to 6.78 ng/g lw, with a median of 3.35 ng/g lw. The HCH isomer profile varied between study areas. While β-HCH was generally the dominant isomer, in Agri-VAC, γ-HCH made a significant contribution. No significant differences were detected between areas or in pairwise comparisons (*p* < 0.05; Tables S9 and S10). The interpretation of the R_α/γ_ ratio ([α-HCH]/[γ-HCH]) is based on its value range. A ratio between 4 and 7 indicates the fresh input of technical HCH, while values below or equal to 1 suggest the source is from lindane usage. Ratios above 7 have been identified as a result of long-range transport or recycling of technical HCH. The R_β/(α+γ)_ ratio ([β-HCH]/[α-HCH + γ-HCH]) is used to indicate the timing of HCH and/or lindane use, with a value below 0.5 suggesting recent usage (Mössner et al. 1992). Based on the R_α/γ_ values, lindane was the dominant component in most samples, with technical HCH observed only in the woodland area (Table 3). The R_β/(α+γ)_ ratio suggests historical but also recent usage in all study areas (Table 3).

The contribution of ∑endosulfan to the OCP profile was minimal, with concentrations ranging from 0.067 to 0.321 ng/g lw and a median of 0.188 ng/g lw, primarily driven by α-endosulfan. Although no significant differences were observed between locations overall (*p* > 0.05; Table S9), pairwise comparisons indicated that Agri-VAC (2.85 ng/g lw) had significantly higher concentrations compared to the woodland area (0.610 ng/g lw) (*p* < 0.05; Table S10).

**Fig. 7:**
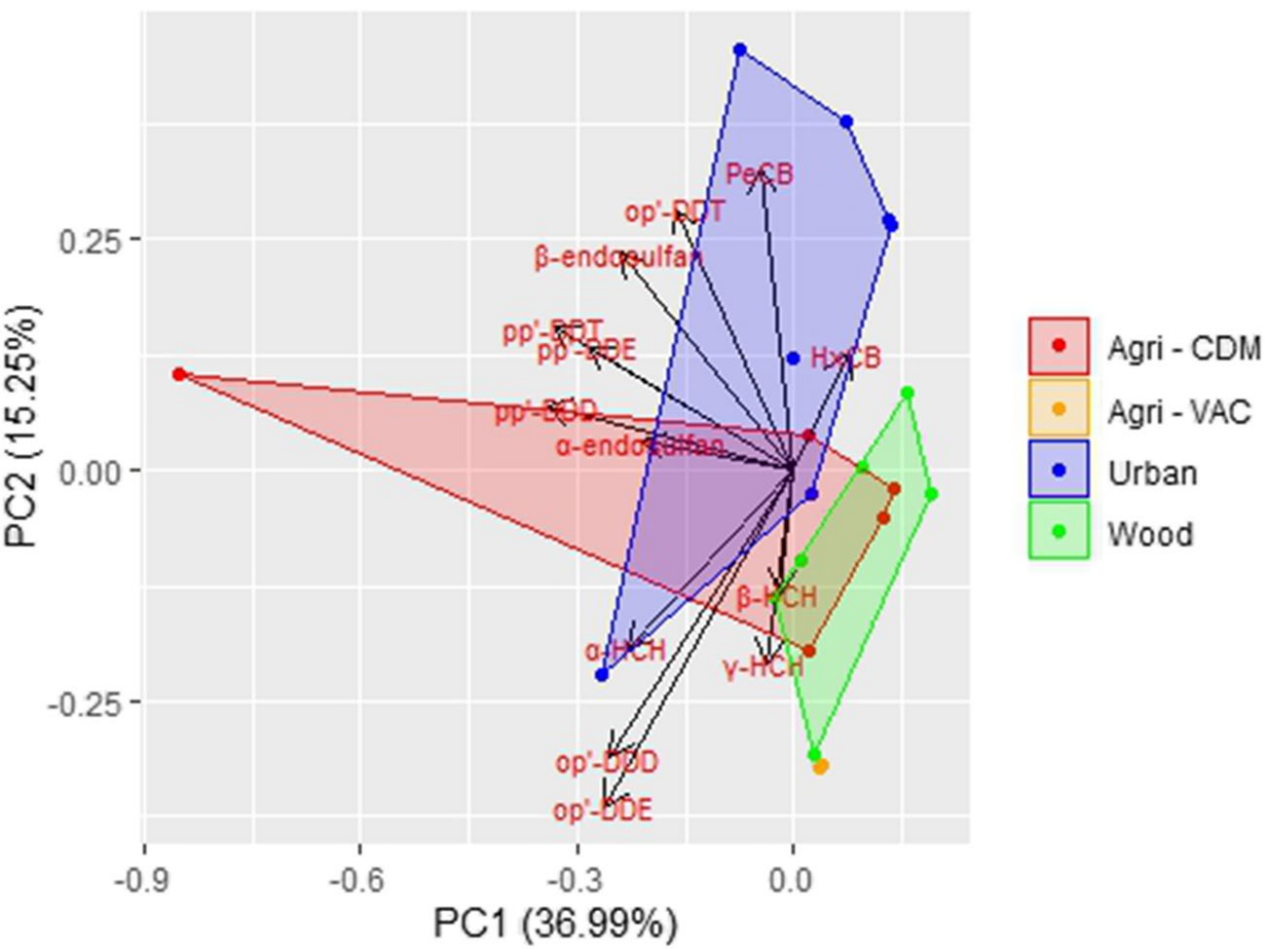
Score plot of OCPs (ng/g lw) in great tit eggs from the different studied areas in the Veneto Region, Italy

In the score plot of different OCP compounds across various locations (on a lw basis), the KMO measure of sampling adequacy (MSA) of 0.51 indicates an acceptable usefulness of the PCA, which summarizes the variability in the data associated with the different studied areas (Fig. 7, Table S11). The first two principal components, PC1 and PC2, together explain 52.64% of the total variability (36.99% and 15.25%, respectively). The studied areas showed a clear separation between urban and woodland areas, while agricultural areas overlapped with the other two. When OCP concentrations were expressed on a ww basis, the KMO value increased to 0.81, indicating a notably stronger sampling adequacy (Table S12). In this case, PC1 and PC2 explained 70.69% and 9.51% of the variability, respectively (Figure S5). The PCA revealed a broader spread of urban samples along both components, while agricultural and woodland samples overlapped more extensively. Compounds within PC1 followed similar distribution trends, but variability was more evident along PC2. These differences highlight how the choice of lw or ww can influence the spatial patterns observed in multivariate analyses. In this context, the PCA based on ww data can be seen as a complementary assessment that reduces the influence of differences in lipid content across eggs, while the lipid-normalized dataset remains the ecotoxicologically relevant basis for interpreting exposure patterns (Van den Steen et al. 2009, 2008).

## Discussion

Great tit eggs were sampled across different environments around Padua to assess contaminant patterns. In this species, laying order had little influence on contaminant concentrations (see Figure S6), consistent with previous observations for OCPs, despite the large clutch size (Van den Steen et al. 2009). Similarly, limited effects of laying order have been reported for PFAS in some passeriform species, though not in great tits, reinforcing its species-specific variability (Morganti et al. 2021).

PFAS have been identified in bird eggs across many regions worldwide. Nonetheless, as far as we are aware, great tit eggs have primarily been used as bioindicators of pollution in areas with fluorochemical industry activity (Groffen et al. 2024, 2019; Lopez-Antia et al. 2021; Morganti et al. 2021). The most likely sources for PFAS in human-impacted areas without emission from prominent hot-spots are direct release from consumer products, small- to medium-scale manufacturing processes, or wastewater treatment plants (Dasu et al. 2022).

In the present study, PFOS was the predominant compound in the PFAS profile of great tit eggs from most of the studied areas. This pattern has been previously observed in other studies utilizing terrestrial bird eggs as bioindicators of diffusive environmental pollution. PFOS was the predominant compound in starling eggs from five different clutches laid in a rural site in Northern Italy, far from any known point source of PFAS release (Morganti et al. 2021). But PFOS was also generally the PFAS present at highest concentration in starling eggs from industrial, rural/agricultural, and landfill locations within five urban centers across Canada (Gewurtz et al. 2018). The widespread presence of PFOS reflects its accumulation through dietary intake and its annual maternal transfer to the eggs (Lopez-Antia et al. 2021). Although major companies phased out PFOS in the early 2000s, PFOS concentrations remain present in our environments (Saini et al. 2023). This could be due to a delayed ecosystem response to changes in atmospheric concentrations, including precursor compounds. More specifically, changes in chemical production likely first impacted atmospheric concentrations, allowing PFAS and precursors already present in the atmosphere to continue being deposited. This persistence can be explained by several factors such as the extensive historical production of PFOS, its extreme chemical stability, the presence of both primary and secondary sources, and its potential for long-range atmospheric transportation. In this context, higher PFOS proportions in some sites (e.g. agricultural areas) likely reflect a stronger influence of historical deposition combined with local retention processes, whereas lower PFOS proportions (e.g. urban areas) suggest either greater dilution from other PFAS sources or reduced historical accumulation. These factors combined may explain why PFOS was still detected despite regulatory efforts (Groffen et al. 2024). After PFOS, PFCAs with an even-numbered carbon chain such as PFDoDA and PFDA showed a relevant contribution on the PFAS profile. This finding was previously observed in great tit eggs from a highly contaminated area in Belgium (Groffen et al. 2024, 2019; Lopez-Antia et al. 2021), but not consistent in all great tit eggs studies (Morganti et al. 2021). Conversely, in marine environments, odd-numbered carbon chain PFCAs were most commonly reported in aquatic bird eggs compared to the homologue even-numbered (Colomer-Vidal et al. 2022; Elliott et al. 2023; Pereira et al. 2021). Some researchers have suggested that these differences may be due to substantial contributions from atmospheric sources, where fluorotelomer-based precursor compounds with even numbers degrade into both odd- and even-numbered PFCAs in comparable amounts. The increasing proportion of odd-numbered PFCAs is then thought to be driven by higher bioaccumulation of longer chain odd-numbered PFCAs (Eriksson et al. 2016; Lu et al. 2024). This mechanism is consistent with the elevated odd-numbered PFCAs observed in our woodland site, which is relatively isolated from local emissions and therefore more likely to reflect long-range atmospheric transport.

∑PFAS concentrations observed in the present study were similar to those reported in great tit eggs from the Po River valley in northern Italy (Morganti et al. 2021). The Po River flows through different Italian regions, including the Veneto region, where the present study was conducted. However, it is important to note that the Po River does not pass directly through the sampling sites of this study, and these are not included in the well-known contaminated sites of Veneto. The presence of these compounds in the study areas therefore highlights widespread contamination from diffuse sources, with varying influences depending on the environment type. Such diffuse contamination can arise from a mix of atmospheric deposition, surface water transport, and localized inputs from human activities, each influencing the relative proportions of PFAS compounds detected. PFAS concentrations reported in the present study were much lower than those reported in great tit eggs from Belgium (Groffen et al. 2024, 2019; Lopez-Antia et al. 2021) in studies conducted to assess PFAS accumulation in great tit eggs near a fluorochemical plant operated by 3M. PFOS concentrations detected in the vicinity of the plant were up to four orders of magnitude higher than those found in the present study (see Table S13). Similarly, great tit eggs showed higher concentrations of ∑PFCAs in the surrounding of the fluorochemical plant compared to this study area; however, further away from the plant, PFCA concentrations are of the same order of magnitude between the two study areas (Groffen et al. 2024).

Although no statistically significant differences were observed in the ∑PFAS contamination concentrations in great tit eggs collected from the different areas, distinct variations in contamination by specific compounds were evident (Fig. 3). High concentrations of PFOS at the Agri-CDM could be attributed to its intrinsic characteristics. Specifically, the site was designed as a catchment basin with the function of removing nitrogen and phosphorus from wastewater originating from upstream intensive agricultural activities before the water is released into the Venice Lagoon. However, the collection of water from surrounding and upstream farmlands may have contributed to the presence of PFOS, potentially introduced through the use of sludge fertilizers in local agricultural activities. Indeed, it has been reported that this type of soil amendment can bind several emerging contaminants, PFAS included (Bertanza et al. 2024; Brambilla et al. 2016). Moreover, this area is not far away from Brenta, Bacchiglione, and Fratta-Gorzone rivers. The Brenta River, which originates in Trentino, flows through Lake Garda and into the Adriatic Sea, merging with the Bacchiglione, which drains the cities of Vicenza and Padua, and the Fratta-Gorzone, which carries treated wastewater from textile and tannery industrial areas, including a fluorochemical factory. Notably, PFOS and PFCAs have been detected in fish samples from Lake Garda, confirming the presence of these contaminants in the river’s sources (Valsecchi et al. 2015). While the Agri-VAC sampling area is also fed by the Brenta River, it does not exhibit such high concentrations of PFOS, though it still may show a contribution. This hydrological connection could facilitate the downstream transport of both PFOS and PFCAs, leading to site-specific differences in composition depending on water flow patterns and retention capacity. The significantly lower concentrations of PFOS observed at the woodland area could be attributed to its distance from both direct and indirect pollution sources.

Great tit eggs from agricultural areas showed PFOS concentrations similar to those from the urban area but had lower concentrations of PFCAs. Conversely, great tit eggs from the woodland area exhibited the lowest PFOS concentrations but similar PFCAs concentrations than those of bird eggs collected in the urban area. Eggs from the urban area displayed balanced concentrations of both PFOS and PFCAs. PFOA concentrations in great tit eggs were generally low across all samples, with the woodland area showing the lowest concentrations of both PFOA and PFOS. Such patterns suggest that PFOS is influenced more by local agricultural and wastewater-linked sources, while PFCAs, particularly the long-chain variants, are more strongly linked to urban-related emissions and/or long-range atmospheric inputs. These contamination patterns are likely influenced by the proximity of PFAS emission sources and their precursors, as well as by processes such as precursor transformations and bioaccumulation mechanisms in living organisms. The PCA (Fig. 4) indicates that PFOS and PFOA in eggs collected from agricultural areas may originate from a common source. The concentrations of PFOS and PFOA detected in great tit eggs from agricultural areas were comparable in magnitude to those measured in eggs collected from urban Padua (Fig. 3). Nevertheless, these agricultural areas are situated far from the urban area of Padua and atmospheric transport and ground fallout of PFOS and PFOA does not appear to be the primary pathway for the environmental contamination of these compounds (Saini et al. 2023). Potential additional sources of PFAS in agricultural areas could include the intentional or unintentional presence of PFAS in pesticides used for farming (Donley et al. 2024) and the application of sewage sludge (biosolid) as a soil amendment (EEA 2024b). While PFOA is not a breakdown product of commonly used pesticide active ingredients, certain fluoropolymers, such as PTFE, are listed as potential inert ingredients in pesticide formulations and might be contaminated with non-polymeric PFAS (Lohmann et al. 2020). Moreover, PFOA and other short-chain PFCAs can leach from fluorinated plastic containers commonly used in the agricultural sector. It is estimated that 20–30% of all hard plastic containers used in agriculture are fluorinated (Bryer 2022), which may contribute significantly to the release of PFCAs during the application of pesticide formulations to agricultural fields (Donley et al. 2024). Land application of biosolids is an effective practice for recycling nutrients and reducing dependence on commercial fertilizers. However, biosolids application to agricultural land tends to increase the concentrations of PFAS along with other anthropogenic contaminants in the soil, leading to long-term contamination. In USA, biosolids are estimated to be the most widespread source of soil PFAS contamination (Venkatesan and Halden 2013), and globally, biosolids have been identified as a major contributor to PFOA and PFOS contamination in surface soils (Brusseau et al. 2020).

In contrast to PFOS, PFCA concentrations are lower in eggs from the agricultural areas. Regarding PFCAs, the highest concentrations are found in urban and woodland areas for most compounds. The city of Padua is not as directly impacted by PFAS contamination as other areas in the Veneto region. However, it is included in the municipalities with a mild PFAS impact, where a monitoring program has been established to gain a clearer understanding of the extent of exposure (Ingelido et al. 2020; Menegatto et al. 2022). Among the potential PFAS contamination sources, the city is affected by activities related to the airport, as well as waste management operations, including sewage treatment and the disposal of non-hazardous wastes (Luimes and Horel 2024). Such activities could potentially release a broader spectrum of PFAS, including long-chain PFCAs, which might help explain the relatively higher proportions of even-chain PFCAs observed in urban eggs. The PFCA concentrations in great tit eggs collected from the woodland area, located 15 km from Padua at an elevation of 300–400 m above sea level, are of the same order of magnitude as those found in eggs collected near the city of Padua. However, eggs from the woodland area exhibit a reversed odd–even pattern compared to those from the urban and agricultural areas (Fig. 3), a trend also observed in the PCA analysis (Fig. 4). In terrestrial locations far from local emission sources, the occurrence of PFCAs is dominated by long-range transport of volatile PFCA precursors, such as fluorotelomer alcohols (FTOH), which are then oxidized to produce PFCAs (Rankin et al. 2016) and the capture of PFCAs precursors by plants, which determines their availability along the food chain (Wang et al. 2020). In fact, deciduous plant leaves effectively capture semi-volatile PFAS that adhere to their surfaces. When the leaves fall and decompose, they transfer these captured PFAS into the soil (Wang et al. 2020). Through the food web, PFAS in the soil can be transferred to invertebrates and then to their avian predators. Studies have shown that PFAS concentrations found in invertebrates, such as earthworms (Navarro et al. 2016) and isopods (Groffen et al. 2019), can serve as reliable proxies for those found in the tissues of their avian predators such as great tits (Wang et al. 2020). In eggs collected from the forested area of the woodland area, even-chain PFCAs exhibit lower concentrations compared to their longer odd-numbered counterparts (e.g., PFDA < PFUnDA, PFDoDA < PFTrDA). This pattern supports the understanding that atmospheric oxidation and microbial degradation fluorotelomer-based precursor compounds with even numbers of carbon atoms, e.g., fluorotelomer alcohols (FTOH), yield equimolar even-numbered and odd-numbered PFCAs along with lower yields of shorter chain homologues (Ellis et al. 2004; Wallington et al. 2006). Previous studies on biota from remote, minimally human-impacted areas (Elliott et al. 2023; Sturm and Ahrens 2010) suggest that the increasing proportion of odd-numbered PFCAs is likely due to their higher persistence and bioaccumulation potential of these longer chain homologues (Lu et al. 2024). In contrast, eggs from urban and agricultural areas showed higher concentrations of even-chain PFCAs (PFDA and PFDoDA) compared to their corresponding longer odd-chain PFCA with one additional carbon atom (PFDA > PFUnDA, PFDoDA > PFTrDA). This likely reflects the influence of local emission profiles, urban and agricultural areas receiving a larger contribution from sources that preferentially generate even-numbered homologues, combined with atmospheric chemistry under elevated NOx conditions that further favors even-chain PFCA formation, as observed in regions with high NOx concentrations typical of urban and the studied agricultural areas, located in a flat region with a dense road network (Ellis et al. 2004; Wallington et al. 2006).

The OCP profile observed in this study was largely driven by the DDT family, with p,p′-DDE distinctly dominant. Other DDT compounds, including p,p′-DDT, were present at much lower concentrations. The dominance of DDTs, and specially p,p′-DDE, over other OCPs families has been previously reported in studies focused on great tit eggs (Van den Steen et al. 2009). These previous mentioned studies collected samples from the early 2000s, and nearly two decades later, this pattern remains unchanged. This distinctive pattern has also been reported in the eggs of other bird species such as white stork, red kite, purple heron (Roscales et al. 2017), black-crowned night-heron, little egret (Fasola et al. 1998), and yellow-legged gulls (Zapata et al. 2018). In our study, HCB and, subsequently, ∑HCHs were the next contributors to the ∑OCPs profile, a trend consistent with previous studies on great tit eggs.

The concentrations detected in the present study were generally within the same order of magnitude as those previously reported (as shown in Table S14). However, while the concentrations of most OCPs with comparable data in the literature were slightly higher in the literature, especially for HCB and HCHs (Van den Steen et al. 2009, 2008), the concentration of p,p′-DDE was notably higher in the present study.

The concentrations of p,p′-DDE, and consequently ∑DDTs and ∑OCPs, found in Agri-CDM and urban areas were significantly higher than in woodland areas and Agri-VAC. The elevated concentrations at Agri-CDM are likely a result of its position as an artificial phytoremediation site that receives wastewater from upstream areas. Surrounded by intensive agricultural activities, it is situated near the Bacchiglione and Brenta rivers, both affected by diffuse agricultural pollution and point-source contamination from urban and industrial wastewater discharges (Bertanza et al. 2024; Brambilla et al. 2016; Masiol et al. 2018). Moreover, Agri-CDM is located near the Lagoon of Venice, which was heavily contaminated by organic pollutants from historical industrial, agricultural, and urban discharges. High concentrations of OCPs like DDT and HCH, mainly from agricultural runoff and tributary drainage, pose a serious threat to the Lagoon’s biodiversity and ecosystem stability (Parolini et al. 2010). Additionally, p,p′-DDT concentrations were significantly higher in urban areas compared to woodland areas. The observed concentrations and differences across sampling sites were attributed to the historical use of DDT in the Veneto region. Urban areas may exhibit higher concentrations of OCPs like DDT compared to agricultural or woodland areas, possibly because of its more extensive historical use for controlling pests in cities. Unlike agricultural soils, urban soils tend to be less regularly disturbed and more compacted by leaf litter and mulch. This reduced disturbance could contribute to limiting the dilution and degradation of persistent compounds, allowing DDT to remain concentrated in the soil. These urban soil reservoirs might therefore act as long-term sources, reflecting the notable persistence of DDT in the environment (Hites and Venier 2023). Previous studies have shown greater variability in ∑OCPs concentrations and profiles, suggesting that local contamination sources such as urbanization, industrialization, and agriculture play a significant role in ∑OCPs (p,p′-DDE up to 92%) concentrations. Notably, significantly higher ∑OCPs concentrations were detected in rural sampling locations situated within residential areas (Van den Steen et al. 2008). Another study compared several locations across Europe and concluded that ∑OCPs concentrations (p,p′-DDE up to 90%) were significantly higher in rural areas associated with agricultural activities than in remote and urban areas, following the order: rural > remote > urban (Van den Steen et al. 2009). In this context, the observed ∑OCPs concentrations were higher in urban and agricultural areas compared to woodland areas. This pattern reflects the historical use of OCPs in urban pest control and agricultural activities, as well as the limited soil disturbance in urban environments, which promotes the persistence of these compounds. The results emphasize the influence of land use and historical contamination on ∑OCPs distribution in the Veneto region.

However, it is important to note that the ratios calculated in this study indicate that technical DDT predominated in all samples, reflecting its historical use. Since p,p′-DDE is the primary aerobic breakdown product of p,p′-DDT, the accumulation profile of DDTs suggests a historical input rather than contributions from recent sources (Van den Steen et al. 2009). Although DDT and its metabolites have been banned in Europe since the 1980 s, they can still be found in the environment and biota. p,p′-DDT, the principal compound of the technical DDT mixture, was detectable in most egg samples, consistent with previous studies (Van den Steen et al. 2008). The bibliography on OCPs in birds from this area and Northern Italy is limited. Despite the long-standing ban on DDT in Italy, it has remained detectable in bird eggs (little egret and black-crowned night-heron) in the region, persisting from decades ago to the present (Fasola et al. 1998). Similarly, studies from Southern Italy have highlighted the persistent presence of DDTs in bird eggs and liver from both aquatic and terrestrial environments (Naso et al. 2003; Provini and Galassi 1999), suggesting that DDTs may still be used for mosquito control or in agricultural practices in the Mediterranean, despite legal restrictions (Provini and Galassi 1999). While p,p′-DDE concentrations in bird eggs have gradually declined since the 1980s, recovery has been slow, emphasizing the long-lasting impact of historical contamination. This slow recovery is also reflected in the persistence of DDTs in areas such as the Veneto Region, where ∑DDTs were detected in alpine air samples (Kirchner et al. 2016). A study based in Lake Iseo (Northern Italy) observed a significant increase in p,p′-DDE concentrations from the mid-1990s, long after the ban, likely linked to the recent retreat of glaciers in northern Italian lakes, which began in 1990 and released old pesticides stored in the ice during their historical agricultural use (Bettinetti et al. 2011). In contrast, the ratios for HCHs showed a less precise origin, as both recent and historical use were reflected in our data. Specifically, lindane (also known as γ-HCH) primarily contributes to the presence of HCHs in this study, with occasional fresh inputs of technical HCHs observed in woodland areas. Similar to DDT, lindane has remained detectable in bird eggs from the region long after its ban (Fasola et al. 1998), in contrast to studies in birds from Southern Italy, where HCHs were barely detected (Naso et al. 2003; Provini and Galassi 1999). Lindane has been previously detected in air samples from alpine regions influenced by the Veneto Region and the Po Valley (Kirchner et al. 2016), in water and fish from the Po River Delta (Provini and Binelli 2006), and his prevalence have been highlighted in sediments from the Lagoon of Venice (Parolini et al. 2010), as well as in studies demostrating the utility of bioindicators for monitoring environmental contamination. OCP contamination reflects both past and recent inputs, with DDT and its metabolites, particularly p,p′-DDE, persisting in urban soil due to limited disturbance, creating long-term reservoirs. Higher p,p′-DDE concentrations in urban areas compared to rural or woodland areas underscore the lasting impact of historical pesticide use in cities. In contrast, HCHs, especially lindane, show evidence of both historical application and recent agricultural contributions, with higher concentrations detected in woodland areas. Geographical differences reveal that urban areas exhibit significantly higher OCP concentrations, consistent with trends across Italy and Europe, while local sources such as agricultural runoff and industrial discharges shape the contamination profiles near rivers and industrial zones.

Despite regulatory bans, the persistence of OCPs underscores the need for continued monitoring, as contamination concentrations have remained largely unchanged over the past two decades. Similarly, PFAS contamination demonstrates the complexity of diffuse sources and environmental pathways. The use of great tit eggs as bioindicators revealed that while total PFAS concentrations were comparable across forested, urban, and agricultural areas, the profiles of individual compounds varied, reflecting distinct contamination patterns. PFOS was consistently the predominant compound, highlighting its widespread persistence despite regulatory phase-outs. Forested areas showed PFAS contamination likely influenced by long-range atmospheric transport and capture and adsorption by vegetation surfaces, while agricultural areas exhibited unique patterns potentially linked to biosolid applications and pesticide use. Urban areas displayed a more balanced PFOS and PFCA profile, suggesting localized emissions. The odd–even PFCA patterns observed across locations further emphasize the role of atmospheric chemistry, particularly the oxidative degradation of PFAS precursors, in shaping contamination profiles. These findings underscore the importance of addressing both historical and ongoing sources of contamination while integrating bioindicators like great tit, providing a potentially non-invasive method of environmental monitoring. This approach is essential for unraveling the complex sources and pathways of OCP and PFAS contamination across diverse landscapes in the Veneto region.

## Supplementary Information

Below is the link to the electronic supplementary material.

ESM 1 (DOCX 6.79 MB)

## Data Availability

The datasets used and/or analyzed during the current study are available from the corresponding author on reasonable request.
