# Supplementary material for: Evaluation of per- and polyfluoroalkyl substances and organochlorine pesticides in great tit eggs from areas with different anthropogenic pressures in Padova, Italy
Source: Environ Sci Pollut Res Int. 2025 Nov 14;32(46):26240–58. doi: 10.1007/s11356-025-37052-5 (PMC12672631; doi:10.1007/s11356-025-37052-5)
Supplement: Supplementary file 1 — (DOCX 6.79 MB) [file 11356_2025_37052_MOESM1_ESM.docx]

**Evaluation of per- and polyfluoroalkyl substances and organochlorine pesticides in great tit eggs from areas with different anthropogenic pressures in Padova, Italy**

Pere Colomer-Vidal^1^, Juan Muñoz-Arnanz^1^, Laura Giovanetti^2^, Ilaria Caliani^2^, Stefania Ancora^2^, Matteo Schiavinato^3^, Flavio Monti^4^, Matteo Beccardi^5^, Marianna Rusconi^6^, Sara Valsecchi^6^, Silvia Casini^2^, Begoña Jiménez^1^

^1^ Department of Instrumental Analysis and Environmental Chemistry, Institute of Organic Chemistry (IQOG-CSIC), Juan de la Cierva 3, 28006 Madrid, Spain.

^2^ Department of Physical, Earth and Environmental Sciences, University of Siena, 53100 Siena, Italy.

^3^ Department of Toxicology, Wageningen University, 6708 WE Wageningen, The Netherlands.

^4^ Institute of Research on Terrestrial Ecosystems (IRET), National Research Council (CNR), Campus Ecotekne, 73100 Lecce, Italy.

^5^ Institute of Avian Research, An Der Vogelwarte 21, 26386 Wilhelmshaven, Germany.

^6^ Water Research Institute (IRSA), National Research Council (CNR), via Mulino 19, 20861 Brugherio MB, Italy.

Corresponding author: Pere Colomer-Vidal (pere.colomer@iqog.csic.es)

Text S1. Detail of the analytical considerations and procedures

*PFAS*

LC–MS grade acetonitrile, LC–MS grade methanol, concentrated formic acid, ammonium hydroxide (25% in water) and ammonium acetate were purchased from Sigma-Aldrich (Missouri, US). All other reagents were at least analytical reagent grade. Clean water (DQ, 18.2 MΩcm resistivity) was produced by a Millipore Direct-QUV water purification system (Millipore, Massachusetts, US). Certified mixture solution containing C6-C14 perfluoroalkyl carboxylates (PFCA), C4-C6-C8-C10 perfluoroalkyl sulfonates (PFSA), perfluorooctane sulfonamide (FOSA) and 6:2, 8:2 fluorotelomer sulfonates (FTS), ADONA (CAS 958445-44-8) and HFPO-DA (CAS 62037-80-3; also known as GenX) were purchased by (Wellington Laboratories, Ontario, Canada). Analytical standard of perfluoro([5-methoxy-1,3-dioxolan-4-yl]oxy)acetic acid (C6O4) (P5MeODIOXOAc, CAS Number 1190931-41-9) was supplied by Ultra Scientific (Italy) on the behalf of Solvay Specialty Polymers (Bollate, Italy). Commercial certified native solutions and methanolic stock solutions of C6O4 were diluted in methanol to obtain mixed native standard solutions at different concentration. Mass-labelled MPFAC-MXA solution (Wellington Laboratories) and mass-labelled d-N-EtFOSA-M solution (Wellington Laboratories) were diluted in methanol (40 µg/L) for the preparation of the stable isotope labelled solution used as internal standard mixture (SIL-IS). Details on the analyte names and abbreviations are reported in Table S2.

First the eggs were cleaned externally, measured and weighed. Eggs were then opened and the fresh weight of their content was recorded (about 0.6 -1 wet wt). Afterward the egg content was freeze-dried at -20°C and again weighed to record dry weight.

Great tit eggs were extracted according to Mazzoni et al., (2016). The dry content of the egg (0.1-0.2 g dry wt) were weighted into a PP centrifuge tube and spiked with 100 µl of 40 µg/L of SIL-IS solution. 5 mL of water and acetonitrile solution (10:90 v/v) were added to the solid sample. 70 µL of formic acid were added to the extraction mixture and vigorously shaken. Subsequently the tube was immersed in an ultrasonic bath for 15 min and then centrifuged for 12 min at 8,000 rpm at 10 °C. The extraction was repeated twice and the combined supernatants were transferred in a new 50 mL PP tube where 2 g MgSO4 and 0.5 g NaCl were added. The tube was immediately shaken to prevent coagulation of MgSO4, centrifuged and stored at −4 ◦C for one night.

The surnatant of all samples was reduced to 1 mL under a gentle nitrogen stream. To remove phospholipids, volume reduced extracts (1 mL) of egg samples were acidified (50 µL formic acid) and filtered through HybridSPE®Phospholipid Ultra cartridges (30 mg, 1 mL SPETubes, Sigma Aldrich), previously cleaned with 3 mL of acetonitrile and 50 µL of formic acid.

*OCPs*

Throughout all stages related to the analytical treatment of the egg samples, the materials used (glass, plastic, or metal) were thoroughly cleaned before use. They were soaked overnight in Nuclean soap (National Diagnostics, Georgia, USA, specialized for the removal of isotopically labeled compounds), then rinsed, dried with acetone, and washed thrice with each of the following solvents in decreasing order of polarity: acetone, dichloromethane, and n-hexane.

For extracting the target analytes, a protocol employing ultrasonication was followed. Approximately 0.2 g of lyophilized egg material was homogenized with 1.5 g of anhydrous sodium sulfate in a mortar and introduced into a 25 mL cylindrical flask. Then, they were fortified with 15 µL of 13C-organochlorine pesticides were added (namely, 13C6-PeCB, 13C6-HCB, 13C6-α-HCH, 13C6-ɣ-HCH, 13C10-p,p-DDE, 13C10-o,p’-DDT, 13C10- p,p’-DDT, 13C9-α-endosulfan and 13C9-β-endosulfan). After fortification, 10 mL of a cyclohexane:acetone mixture (3:1, v/v) was added. The flask containing the solvent and sample underwent sonication in an ultrasonic bath (J.P. Selecta, Barcelona, Spain) at room temperature for 15 minutes. This sonication process was repeated three times, changing the solvent between each cycle, yielding a final volume of approximately 30 mL in a 200 mL glass cell of a TurboVap® evaporation system (Zynmarck Inc., Hopkinton, Massachusetts, USA). Subsequently, the extract was concentrated to a final volume of around 1 mL using nitrogen at 0.8 bars and a temperature of 40°C in the TurboVap® system. The final volume was transferred to a 15 mL Falcon tube along with a wash of the TurboVap® cell performed with cyclohexane:ethyl acetate (1:1, v/v) and brought up to 5 mL using the same solvent mixture. Samples were centrifuged at 2000 rpm for 5 min at room temperature, and the supernatant was transferred to a 15 mL cylindrical glass cell.

The purification stage aimed to separate lipids and/or impurities co-extracted with the target analytes in the previous step. To ensure maximal removal of undesired compounds, two distinct purification procedures were conducted for each sample. The first utilized the GPC uno system (LCTech GmbH, Dorfen, Germany), an automatic gel permeation chromatography system (GPC). The elution of the sample employed a cyclohexane:ethyl acetate solvent mixture (1:1, v/v), injecting the 5 ml of the extract obtained in the previous step into the equipment using a glass syringe. The process lasted 55 min, collecting three different fractions. Only the first two fractions were collected in TurboVap® cells. Fraction I (110mL) contained lipids and larger molecules, fraction II (110 mL) contained the target analytes, and fraction III (40 mL) corresponded to column cleaning and was discarded. Both fractions (I and II) were concentrated to 1 mL using the TurboVap® system. The milliliter from fraction II was transferred to a beaker along with two washes of the cell using n-hexane:dichloromethane (9:1, v/v) and brought up to a volume of 4 mL using the same mixture, which was then divided into two aliquots. One mL was collected in a vial (previously heated at 400°C to ensure the absence of any target analytes), and used for pesticide content analysis. The other 3 ml were collected in a TurboVap® vessel and concentrated to 1 mL. This resulting milliliter from the second aliquot underwent re-purification by adsorption chromatography using a 10 mL glass column, packed with 1.5 g of 44% weight-modified acidic silica with sulfuric acid and an approximate amount of granular anhydrous sulfate both below and above the silica. After conditioning the column with 8 mL of n-hexane:dichloromethane (9:1, v/v), the sample was loaded and eluted with 10 mL of the same solvent mixture. The purified eluate was collected in a TurboVap® cell, concentrated to 1 mL, and then transferred to a vial. As a result, each sample yielded one vial containing 1 mL. Additionally, fraction I containing lipid content was collected in a pre-weighed topaz vial using a precision balance.

The contents of the vials were evaporated to dryness using nitrogen at 40°C with a Pasvial apparatus (Model V3, HiTC. S.A., Spain). Once dried, the vials were reconstituted with 10 µL of PCB IS and 10 µL of n-nonane. After reconstitution, the vials were vortexed and stored in a freezer until instrumental analysis. Simultaneously, the vial with fraction I was concentrated using Pasvial until complete solvent evaporation. It was then placed in an oven at 105°C for 30 min. After cooling to room temperature overnight, the vial was reweighed on a precision balance to determine the weight of the extracted organic matter, representing the lipid content of the samples.

The limits of detection (LOD, pg/g lw) for the target compounds were as follows: PeCB (0.075), HxCB (0.10), α-HCH (0.225), β-HCH (0.20), γ-HCH (0.225), α-endosulfan (0.25), β-endosulfan (0.25), o,p′-DDE (0.175), p,p′-DDE (0.15), o,p′-DDD (0.15), p,p′-DDD (0.725), o,p′-DDT (0.25), and p,p′-DDT (0.45). Recovery values for the isotope-labelled standards ranged from 40% to 108%.


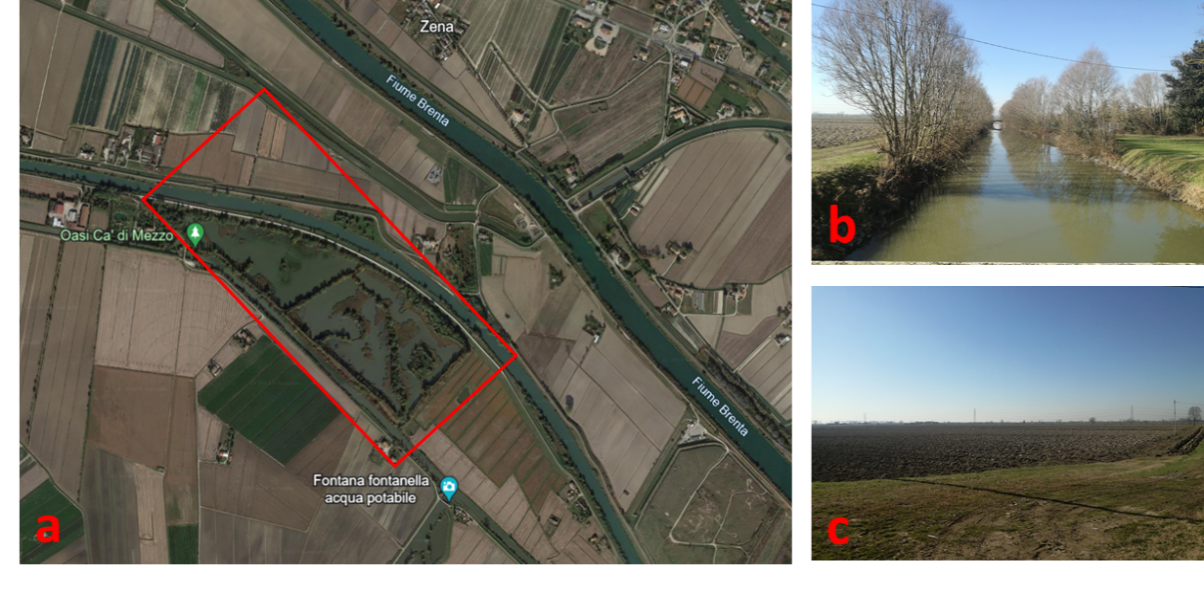


Figure S1. Sampling area of Cà di Mezzo Oasis (Agri - CDM; a), details of the canal that drains water from upstream farmland areas (b) and of the land cover surrounding the study area (c) where the nest boxes are located.


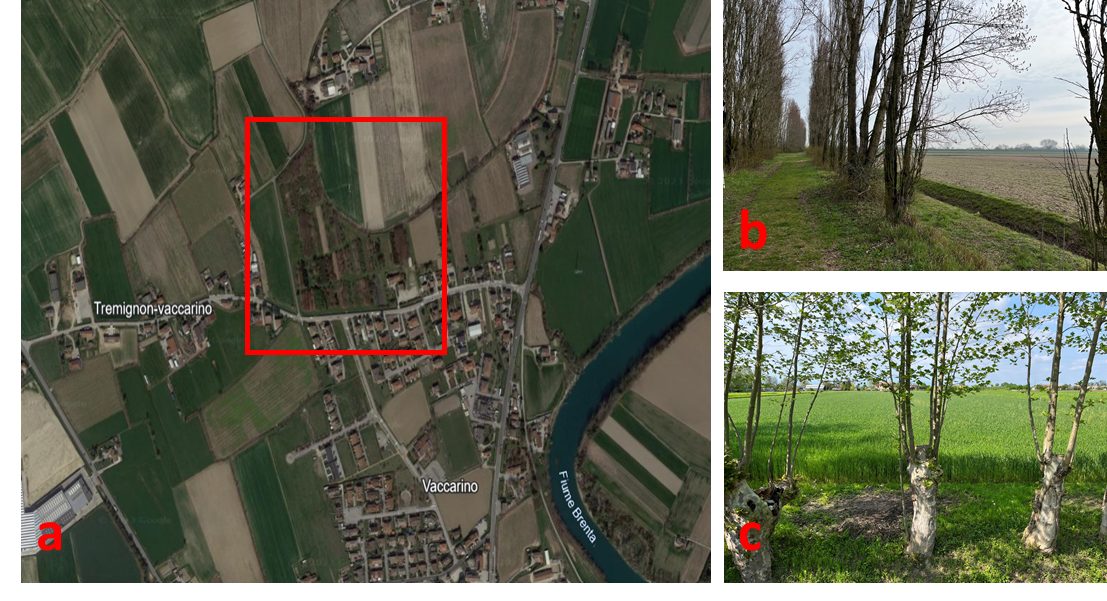


Figure S2. Sampling area of Vaccarino (Agri – VAC; a), details of the land cover surrounding the study area (b, c) where the nest boxes are located.


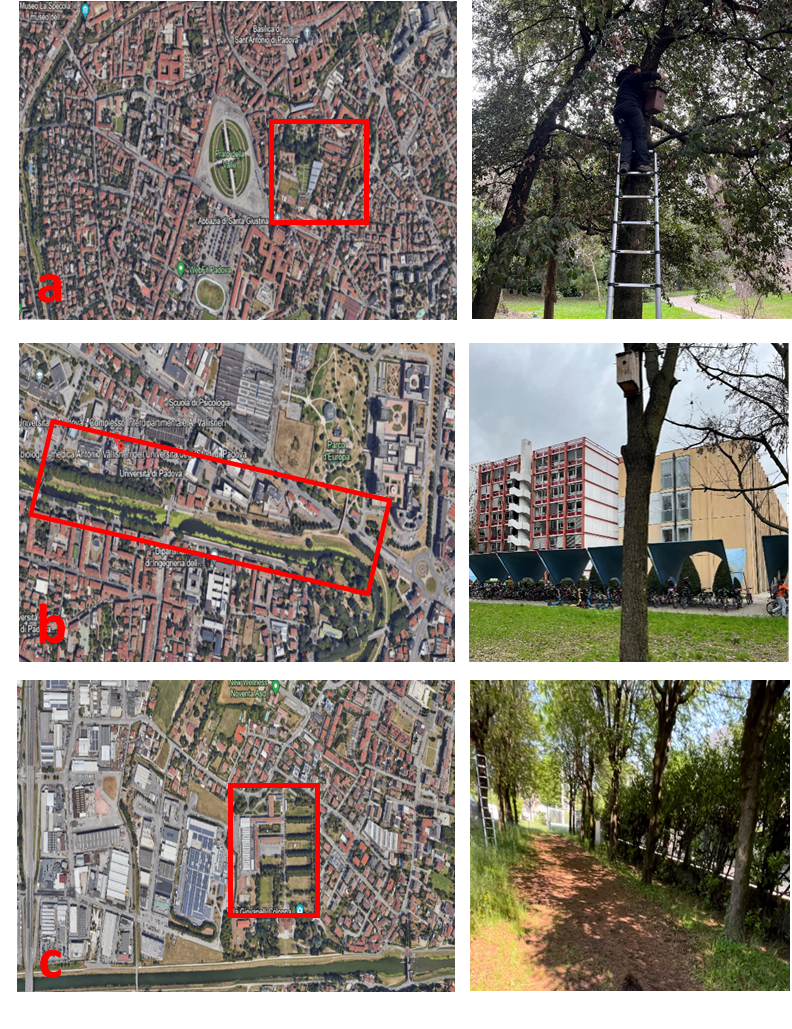


Figure S3. Urban area: Padua’s botanical garden (Urban – OB; a), Vallisneri (Urban – VAL; b), and S. Antonio Village - Noventa Padovana (Urban – VSA; c) where the nest boxes are located.


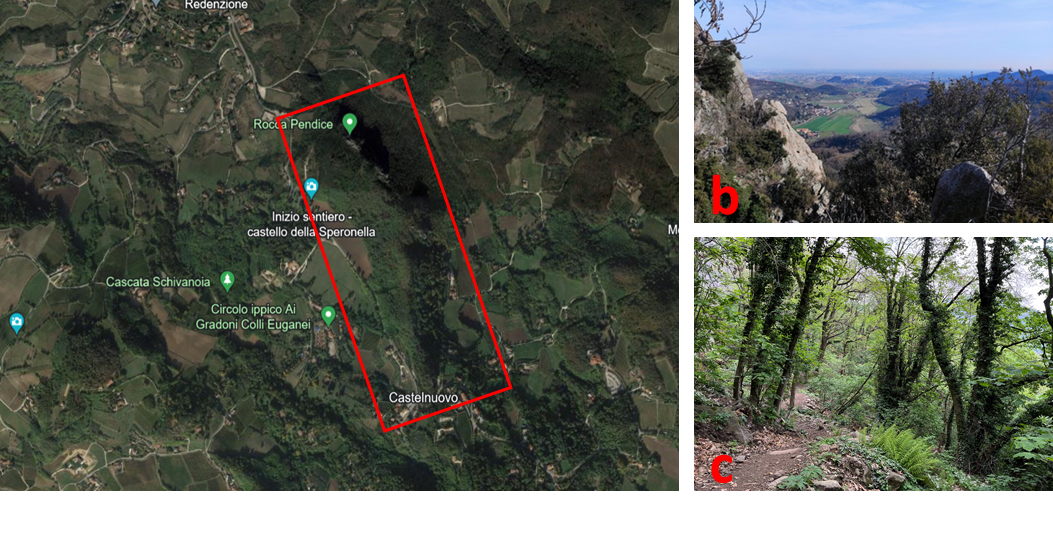


Figure S4. Wood area: sampling area of Euganean Hills Regional Park (Wood – EUG; a), details of the view from the hill (b) and of wood (c) where the nest boxes are located.


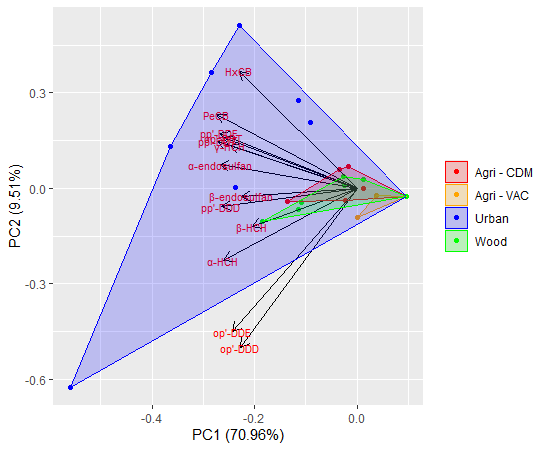


Figure S5. Score plot of OCPs (ng/g ww) in great tit eggs from the different studied areas in the Veneto Region, Italy.
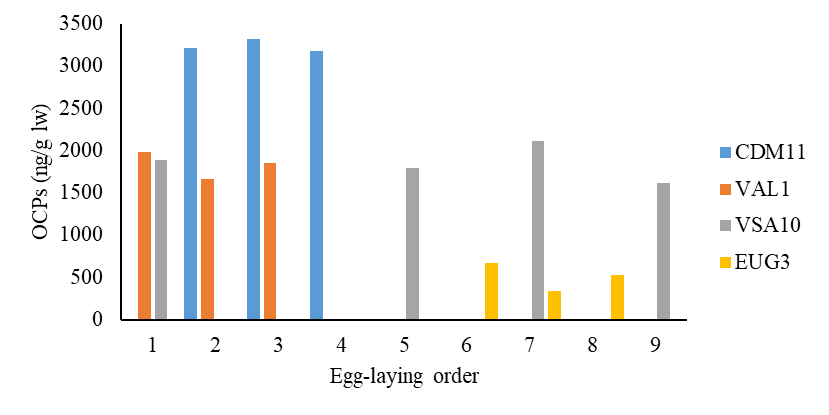

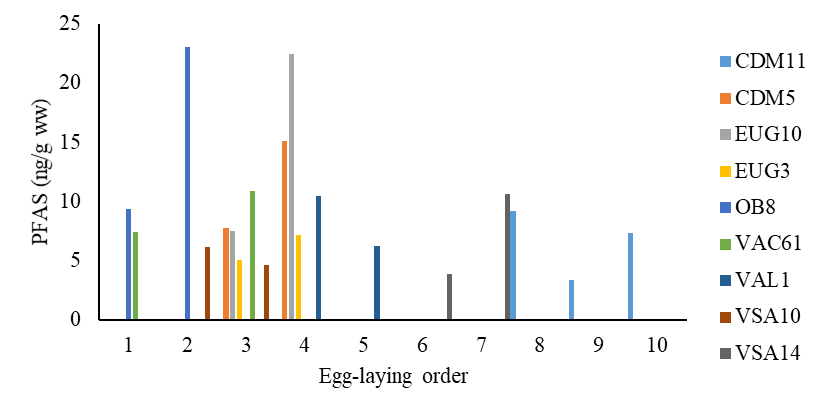


Figure S6. Bar plot of OCPs (ng/g lw) and PFAS (ng/g ww) in great tit eggs to evaluate the effect of laying order in the concentration. Only eggs from the same nest were considered.

Table S1. Details of the great tits’ eggs used in this study.

| **Studied family** | **Year** | **Study area** | **Location name** | **Nest_ID** | **Sample ID** | **Egg status** | **Lipid content (%)*** |
| --- | --- | --- | --- | --- | --- | --- | --- |
| OCPs | 2021 | Urban | Vallisneri | VAL1 | VAL1_1 | Whole | 5.84 |
| OCPs | 2021 | Urban | Vallisneri | VAL1 | VAL1_2 | Whole | 10.29 |
| OCPs | 2021 | Urban | Vallisneri | VAL1 | VAL1_3 | Whole | 15.81 |
| OCPs | 2021 | Urban | Villaggio Sant’Antonio | VSA10 | VSA10_1 | Whole | 15.39 |
| OCPs | 2021 | Urban | Villaggio Sant’Antonio | VSA10 | VSA10_5 | Whole | 14.81 |
| OCPs | 2021 | Urban | Villaggio Sant’Antonio | VSA10 | VSA10_7 | Whole | 9.25 |
| OCPs | 2021 | Urban | Villaggio Sant’Antonio | VSA10 | VSA10_9 | Whole | 12.83 |
| OCPs | 2021 | Woodland | Euganean Hills | EUG10 | EUG10_1 | Whole | 7.84 |
| OCPs | 2021 | Woodland | Euganean Hills | EUG2 | EUG2_1 | Whole | 7.51 |
| OCPs | 2021 | Woodland | Euganean Hills | EUG2 | EUG2_3 | Whole | 4.06 |
| OCPs | 2021 | Woodland | Euganean Hills | EUG3 | EUG3_6 | Whole | 5.53 |
| OCPs | 2021 | Woodland | Euganean Hills | EUG3 | EUG3_7 | Whole | 7.15 |
| OCPs | 2021 | Woodland | Euganean Hills | EUG3 | EUG3_8 | Whole | 4.48 |
| OCPs | 2022 | Agricultural | Cà di Mezzo Oasis | CDM1 | CDM1_1 | Yolk | 2.54 |
| OCPs | 2022 | Agricultural | Cà di Mezzo Oasis | CDM11 | CDM11_2 | Whole | 4.26 |
| OCPs | 2022 | Agricultural | Cà di Mezzo Oasis | CDM11 | CDM11_3 | Whole | 3.92 |
| OCPs | 2022 | Agricultural | Cà di Mezzo Oasis | CDM11 | CDM11_4 | Whole | 4.113 |
| OCPs | 2022 | Agricultural | Cà di Mezzo Oasis | CDM5 | CDM5_1 | Whole | 3.53 |
| OCPs | 2022 | Agricultural | Vaccarino | VAC25 | VAC25_1 | Yolk | 1.75 |
| OCPs | 2022 | Agricultural | Vaccarino | VAC61 | VAC61_2 | Embryo | 3.87 |
| PFAS | 2021 | Urban | Vallisneri | VAL1 | VAL1_4 | Whole | - |
| PFAS | 2021 | Urban | Vallisneri | VAL1 | VAL1_5 | Whole | - |
| PFAS | 2021 | Urban | Villaggio Sant’Antonio | VSA10 | VSA10_2 | Whole | - |
| PFAS | 2021 | Urban | Villaggio Sant’Antonio | VSA10 | VSA10_3 | Whole | - |
| PFAS | 2021 | Urban | Villaggio Sant’Antonio | VSA11 | VSA11_1 | Yolk | - |
| PFAS | 2021 | Woodland | Euganean Hills | EUG10 | EUG10_3 | Whole | - |
| PFAS | 2021 | Woodland | Euganean Hills | EUG10 | EUG10_4 | Whole | - |
| PFAS | 2021 | Woodland | Euganean Hills | EUG2 | EUG2_5 | Whole | - |
| PFAS | 2022 | Agricultural | Cà di Mezzo Oasis | CDM11 | CDM11_10 | Whole | - |
| PFAS | 2022 | Agricultural | Cà di Mezzo Oasis | CDM11 | CDM11_6 | Whole | - |
| PFAS | 2022 | Agricultural | Cà di Mezzo Oasis | CDM11 | CDM11_8 | Whole | - |
| PFAS | 2022 | Agricultural | Cà di Mezzo Oasis | CDM11 | CDM11_9 | Whole | - |
| PFAS | 2022 | Agricultural | Cà di Mezzo Oasis | CDM2 | CDM2_2 | Yolk | - |
| PFAS | 2022 | Agricultural | Cà di Mezzo Oasis | CDM2 | CDM2_3 | Yolk | - |
| PFAS | 2022 | Agricultural | Cà di Mezzo Oasis | CDM5 | CDM5_3 | Whole | - |
| PFAS | 2022 | Agricultural | Cà di Mezzo Oasis | CDM5 | CDM5_4 | Whole | - |
| PFAS | 2022 | Agricultural | Vaccarino | VAC4 | VAC4_1 | Yolk | - |
| PFAS | 2022 | Agricultural | Vaccarino | VAC55 | VAC55_1 | Whole | - |
| PFAS | 2022 | Agricultural | Vaccarino | VAC61 | VAC61_1 | Embryo | - |
| PFAS | 2022 | Agricultural | Vaccarino | VAC61 | VAC61_3 | Embryo | - |
| PFAS | 2022 | Urban | Orto Botanico | OB8 | OB8_1 | Embryo | - |
| PFAS | 2022 | Urban | Orto Botanico | OB8 | OB8_2 | Yolk | - |
| PFAS | 2022 | Urban | Villaggio Sant’Antonio | VSA14 | VSA14_6 | Whole | - |
| PFAS | 2022 | Urban | Villaggio Sant’Antonio | VSA14 | VSA14_7 | Whole | - |
| PFAS | 2022 | Woodland | Euganean Hills | EUG3 | EUG3_3 | Whole | - |
| PFAS | 2022 | Woodland | Euganean Hills | EUG3 | EUG3_4 | Whole | - |
| PFAS | 2022 | Woodland | Euganean Hills | EUG6 | EUG6_1 | Whole | - |
| * Lipid content was only used for OCPs analysis. | | | | | | | |

Table S2. Target analytes measured in the present study including monitoring parent ion, product ions, acronyms, corresponding internal standard (IS) and names of analytes. n/a not applicable.

| **Target analytes** | **Acronym** | **Parent ion (m/z)** | **Product ions (m/z)** | **Internal standard IS** |
| --- | --- | --- | --- | --- |
| Perfluorohexanoate | PFHxA | 312.9 | 119.1, 268.9 | 13C2-PFHxA |
| Perfluoroheptanoate | PFHpA | 362.9 | 169.0, 318.9 | 13C4-PFOA |
| Perfluorooctanoate | PFOA | 412.9 | 169.0, 368.9 | 13C4-PFOA |
| Perfluorononanoate | PFNA | 462.9 | 218.9, 418.9 | 13C5-PFNA |
| Perfluorodecanoate | PFDA | 512.9 | 268.9, 468.9 | 13C2-PFDA |
| Perfluoroundecanoate | PFUnDA | 562.9 | 268.8, 518.8 | 13C2-PFUnDA |
| Perfluorododecanoate | PFDoDA | 612.9 | 318.8, 568.9 | 13C2-PFDoDA |
| Perfluorotridecanoate | PFTrDA | 662.9 | 369.0, 619.0 | 13C2-PFDoDA |
| Perfluorotetradecanoate | PFTeDA | 712.9 | 419.0, 669.0 | 13C2-PFDoDA |
| Perfluorobutane sulfonate | PFBS | 298.9 | 80.2, 99.1 | 13C2-PFHxA |
| Perfluorohexane sulfonate | PFHxS | 398.9 | 80.1, 99.0 | 18O2-PFHxS |
| Perfluorooctane sulfonate | PFOS | 498.9 | 80.3, 99.1 | 13C4-PFOS |
| Perfluorodecane sulfonate | PFDS | 598.9 | 80.0, 99.0 | 13C2-PFUnDA |
| Perfluoro-1-octanesulfonamide | FOSA | 497.9 | 78 | d-N-EtFOSA |
| 2,3,3,3-Tetrafluoro-2-(1,1,2,2,3,3,3-heptafluoroproproxy)propanoate | HPFO-DA, GenX | 329,0 | 169.0, 285,0, 185.0 | 13C4-PFOA |
| perfluoro([5-methoxy-1,3-dioxolan-4-yl]oxy)acetate | C6O4 | 339 | 85.0, 113.0 | 13C4-PFOA |
| dodecafluoro-3H-4,8-dioxanonanoate | ADONA | 377 | 85.0, 251.0 | 13C4-PFOA |
| 1H,1H,2H,2H-perfluorooctanesulphonate | 6:2 FTS | 427 | 407 | 13C4-PFOA |
| 1H,1H,2H,2H-perfluorodecanesulphonate | 8:2 FTS | 527 | 507 | 13C2-PFDA |
| Perfluoro-n-[13C2] hexanoate | 13C2-PFHxA | 314.9 | 269.9 | n/a |
| Perfluoro-n-[13C4] octanoate | 13C4-PFOA | 416.9 | 371.9 | n/a |
| Perfluoro-n-[13C5] nonanoate | 13C5-PFNA | 467.9 | 422.9 | n/a |
| Perfluoro-n-[13C2] decanoate | 13C2-PFDA | 514.9 | 469.9 | n/a |
| Perfluoro-n-[13C2] undecanoate | 13C2-PFUnDA | 564.9 | 519.8 | n/a |
| Perfluoro-n-[13C2] dodecanoate | 13C2-PFDoDA | 614.9 | 569.9 | n/a |
| Perfluoro-n-hexane [18O2] sulfonate | 18O2-PFHxS | 402.9 | 103 | n/a |
| Perfluoro-n-octane [13C4] sulfonate | 13C4-PFOS | 502.9 | 99.1 | n/a |
| N-ethyl-[d5]-perfluorooctane sulfonamide | d-N-EtFOSA | 531 | 169 | n/a |

Table S3. Wilcoxon Signed Rank Test table for assessing statistically significant differences (p < 0.05) in PFAS concentrations between 2021 and 2022.

| **parameter1** | **parameter2** | **statistic** | **p.value** | **method** | **alternative** | **effectsize** | **estimate** | **conf.level** | **conf.low** | **conf.high** | **conf.method** | **n.obs** |
| --- | --- | --- | --- | --- | --- | --- | --- | --- | --- | --- | --- | --- |
| PFOA | Year | 51 | 0.2547 | Wilcoxon rank sum test | two.sided | r (rank biserial) | -0.2917 | 0.95 | -0.6586 | 0.1874 | normal | 26 |
| PFNA | Year | 73 | 0.6027 | Wilcoxon rank sum test | two.sided | r (rank biserial) | 0.1406 | 0.95 | -0.3439 | 0.5660 | normal | 24 |
| PFDA | Year | 72 | 0.8384 | Wilcoxon rank sum test | two.sided | r (rank biserial) | 0.0588 | 0.95 | -0.4102 | 0.5033 | normal | 25 |
| PFUnDA | Year | 65 | 0.5618 | Wilcoxon rank sum test | two.sided | r (rank biserial) | 0.1607 | 0.95 | -0.3370 | 0.5882 | normal | 22 |
| PFDoDA | Year | 85 | 0.3364 | Wilcoxon rank sum test | two.sided | r (rank biserial) | 0.2500 | 0.95 | -0.2349 | 0.6353 | normal | 25 |
| PFTrDA | Year | 42 | 0.8940 | Wilcoxon rank sum test | two.sided | r (rank biserial) | 0.0500 | 0.95 | -0.4632 | 0.5381 | normal | 18 |
| PFTeDA | Year | 45 | 0.5869 | Wilcoxon rank sum test | two.sided | r (rank biserial) | 0.1688 | 0.95 | -0.3728 | 0.6246 | normal | 18 |
| PFOS | Year | 57 | 0.4205 | Wilcoxon rank sum test | two.sided | r (rank biserial) | -0.2083 | 0.95 | -0.6053 | 0.2716 | normal | 26 |
| PFDS | Year | 1 | 1.0000 | Wilcoxon rank sum test | two.sided | r (rank biserial) | 1.0000 | 0.95 | 1.0000 | 1.0000 | normal | 2 |
| ∑PFSAs | Year | 57 | 0.4205 | Wilcoxon rank sum test | two.sided | r (rank biserial) | -0.2083 | 0.95 | -0.6053 | 0.2716 | normal | 26 |
| ∑PFCAs | Year | 96 | 0.1917 | Wilcoxon rank sum test | two.sided | r (rank biserial) | 0.3333 | 0.95 | -0.1424 | 0.6840 | normal | 26 |
| ∑PFAS | Year | 68 | 0.8458 | Wilcoxon rank sum test | two.sided | r (rank biserial) | -0.0556 | 0.95 | -0.4972 | 0.4090 | normal | 26 |

Table S4. Wilcoxon Signed Rank Test table for assessing statistically significant differences (p < 0.05) in OCPs concentrations between 2021 and 2022.

| **parameter1** | **parameter2** | **statistic** | **p.value** | **method** | **alternative** | **effectsize** | **estimate** | **conf.level** | **conf.low** | **conf.high** | **conf.method** | **n.obs** |
| --- | --- | --- | --- | --- | --- | --- | --- | --- | --- | --- | --- | --- |
| ∑OCPs | Year | 23 | 0.0813 | Wilcoxon rank sum test | two.sided | r (rank biserial) | -0.4945 | 0.95 | -0.7953 | 0.0016 | normal | 20 |
| ∑DDTs | Year | 22 | 0.0684 | Wilcoxon rank sum test | two.sided | r (rank biserial) | -0.5165 | 0.95 | -0.8059 | -0.0279 | normal | 20 |
| o,p'-DDE | Year | 28 | 0.5259 | Wilcoxon rank sum test | two.sided | r (rank biserial) | -0.2000 | 0.95 | -0.6507 | 0.3549 | normal | 17 |
| p,p'-DDE | Year | 22 | 0.0684 | Wilcoxon rank sum test | two.sided | r (rank biserial) | -0.5165 | 0.95 | -0.8059 | -0.0279 | normal | 20 |
| o,p'-DDD | Year | 35 | 0.7508 | Wilcoxon rank sum test | two.sided | r (rank biserial) | 0.1111 | 0.95 | -0.4432 | 0.6040 | normal | 16 |
| p,p'-DDD | Year | 50 | 0.7513 | Wilcoxon rank sum test | two.sided | r (rank biserial) | 0.0989 | 0.95 | -0.4173 | 0.5668 | normal | 20 |
| o,p'-DDT | Year | 66 | 0.1130 | Wilcoxon rank sum test | two.sided | r (rank biserial) | 0.4505 | 0.95 | -0.0581 | 0.7735 | normal | 20 |
| p,p'-DDT | Year | 61 | 0.2346 | Wilcoxon rank sum test | two.sided | r (rank biserial) | 0.3407 | 0.95 | -0.1865 | 0.7155 | normal | 20 |
| ∑HCHs | Year | 29 | 0.2048 | Wilcoxon rank sum test | two.sided | r (rank biserial) | -0.3626 | 0.95 | -0.7276 | 0.1622 | normal | 20 |
| α-HCH | Year | 39 | 1.0000 | Wilcoxon rank sum test | two.sided | r (rank biserial) | 0.0130 | 0.95 | -0.4999 | 0.5191 | normal | 18 |
| β-HCH | Year | 41 | 0.7513 | Wilcoxon rank sum test | two.sided | r (rank biserial) | -0.0989 | 0.95 | -0.5668 | 0.4173 | normal | 20 |
| γ-HCH | Year | 27 | 0.1538 | Wilcoxon rank sum test | two.sided | r (rank biserial) | -0.4066 | 0.95 | -0.7509 | 0.1116 | normal | 20 |
| ∑endosulfan | Year | 47 | 0.9368 | Wilcoxon rank sum test | two.sided | r (rank biserial) | 0.0330 | 0.95 | -0.4704 | 0.5202 | normal | 20 |
| α-endosulfan | Year | 29 | 0.2908 | Wilcoxon rank sum test | two.sided | r (rank biserial) | -0.3095 | 0.95 | -0.7025 | 0.2281 | normal | 19 |
| β-endosulfan | Year | 0 | 0.2113 | Wilcoxon rank sum test | two.sided | r (rank biserial) | -1.0000 | 0.95 | -1.0000 | -1.0000 | normal | 7 |
| HCB | Year | 54 | 0.5261 | Wilcoxon rank sum test | two.sided | r (rank biserial) | 0.1868 | 0.95 | -0.3404 | 0.6247 | normal | 20 |
| PeCB | Year | 66 | 0.1130 | Wilcoxon rank sum test | two.sided | r (rank biserial) | 0.4505 | 0.95 | -0.0581 | 0.7735 | normal | 20 |

Table S5. Post-hoc statistical power analysis for the Kruskal–Wallis tests assessing differences in PFAS and OCPs concentrations among locations. Effect sizes are expressed as Epsilon-squared (η²) and converted to Cohen’s *f* for power estimation. Power values indicate the probability of detecting a true effect given the observed data.

| Contaminant | η² | *f* | Power values |
| --- | --- | --- | --- |
| ∑PFAS | 0.080 | 0.296 | 0.185 |
| PFOS | 0.388 | 0.796 | 0.891 |
| ∑PFCAs | 0.145 | 0.411 | 0.333 |
| PFOA | 0.099 | 0.332 | 0.225 |
| PFNA | 0.273 | 0.613 | 0.665 |
| PFDA | 0.007 | 0.085 | 0.059 |
| PFUnDA | 0.153 | 0.424 | 0.353 |
| PFDoDA | 0.390 | 0.799 | 0.893 |
| PFTrDA | 0.443 | 0.891 | 0.950 |
| PFTeDA | 0.414 | 0.841 | 0.923 |
| ∑OCPs | 0.889 | 2.826 | 1.000 |
| ∑DDTs | 0.884 | 2.757 | 1.000 |
| o,p’-DDE | 0.131 | 0.389 | 0.162 |
| p,p’-DDE | 0.884 | 2.757 | 1.000 |
| o,p’-DDD | 0.025 | 0.161 | 0.067 |
| p,p’-DDD | 0.329 | 0.700 | 0.456 |
| o,p’-DDT | 0.351 | 0.736 | 0.498 |
| p,p’-DDT | 0.411 | 0.836 | 0.614 |
| ∑endosulfan | 0.076 | 0.287 | 0.108 |
| α-endosulfan | 0.080 | 0.294 | 0.111 |
| ∑HCHs | 0.144 | 0.410 | 0.177 |
| α-HCH | 0.042 | 0.210 | 0.080 |
| β-HCH | 0.286 | 0.633 | 0.380 |
| γ-HCH | 0.248 | 0.574 | 0.318 |
| HCB | 0.400 | 0.816 | 0.591 |
| PeCB | 0.144 | 0.410 | 0.177 |

Table S6. Kruskal–Wallis one-way analysis of variance (p<0.05). In red those that shows significant differences in PFAS concentrations (ng/g ww) among locations. In red are highlighted the statistically significant results.

| **id** | **parameter1** | **parameter2** | **statistic** | **df.error** | **p.value** | **method** | **effectsize** | **estimate** | **conf.level** | **conf.low** | **conf.high** | **conf.method** | **conf.iterations** | **n.obs** |
| --- | --- | --- | --- | --- | --- | --- | --- | --- | --- | --- | --- | --- | --- | --- |
| ∑PFAS | level | Area_type | 2.0104 | 3 | 0.5702 | Kruskal-Wallis rank sum test | Epsilon2 (rank) | 0.0804 | 0.95 | 0.0260 | 1 | percentile bootstrap | 100 | 26 |
| PFOS | level | Area_type | 9.6997 | 3 | 0.0213 | Kruskal-Wallis rank sum test | Epsilon2 (rank) | 0.3880 | 0.95 | 0.1293 | 1 | percentile bootstrap | 100 | 26 |
| ∑PFCAs | level | Area_type | 3.6126 | 3 | 0.3064 | Kruskal-Wallis rank sum test | Epsilon2 (rank) | 0.1445 | 0.95 | 0.0447 | 1 | percentile bootstrap | 100 | 26 |
| PFOA | level | Area_type | 2.4811 | 3 | 0.4787 | Kruskal-Wallis rank sum test | Epsilon2 (rank) | 0.0992 | 0.95 | 0.0399 | 1 | percentile bootstrap | 100 | 26 |
| PFNA | level | Area_type | 6.2840 | 3 | 0.0986 | Kruskal-Wallis rank sum test | Epsilon2 (rank) | 0.2732 | 0.95 | 0.1708 | 1 | percentile bootstrap | 100 | 24 |
| PFDA | level | Area_type | 0.1730 | 3 | 0.9818 | Kruskal-Wallis rank sum test | Epsilon2 (rank) | 0.0072 | 0.95 | 0.0147 | 1 | percentile bootstrap | 100 | 25 |
| PFUnDA | level | Area_type | 3.2033 | 3 | 0.3613 | Kruskal-Wallis rank sum test | Epsilon2 (rank) | 0.1525 | 0.95 | 0.0705 | 1 | percentile bootstrap | 100 | 22 |
| PFDoDA | level | Area_type | 9.3477 | 3 | 0.0250 | Kruskal-Wallis rank sum test | Epsilon2 (rank) | 0.3895 | 0.95 | 0.1571 | 1 | percentile bootstrap | 100 | 25 |
| PFTrDA | level | Area_type | 7.5237 | 3 | 0.0570 | Kruskal-Wallis rank sum test | Epsilon2 (rank) | 0.4426 | 0.95 | 0.2096 | 1 | percentile bootstrap | 100 | 18 |
| PFTeDA | level | Area_type | 7.0446 | 3 | 0.0705 | Kruskal-Wallis rank sum test | Epsilon2 (rank) | 0.4144 | 0.95 | 0.3446 | 1 | percentile bootstrap | 100 | 18 |

Table S7. Dunn’s Test for multiple comparisons (p<0.05). In red those that shows significant differences in PFAS concentrations (ng/g ww) among locations. In red are highlighted the statistically significant results.

| **id** | **group1** | **group2** | **statistic** | **p.value** | **alternative** | **distribution** | **p.adjust.method** | **test** |
| --- | --- | --- | --- | --- | --- | --- | --- | --- |
| ∑PFAS | Agri - CDM | Agri - VAC | 0.0000 | 1.0000 | two.sided | z | None | Dunn |
| ∑PFAS | Agri - CDM | Urban | 0.2018 | 0.8401 | two.sided | z | None | Dunn |
| ∑PFAS | Agri - CDM | Wood | 1.2534 | 0.2101 | two.sided | z | None | Dunn |
| ∑PFAS | Agri - VAC | Urban | 0.1692 | 0.8656 | two.sided | z | None | Dunn |
| ∑PFAS | Agri - VAC | Wood | 1.0803 | 0.2800 | two.sided | z | None | Dunn |
| ∑PFAS | Urban | Wood | 1.1301 | 0.2584 | two.sided | z | None | Dunn |
| PFOS | Agri - CDM | Agri - VAC | 0.7375 | 0.4608 | two.sided | z | None | Dunn |
| PFOS | Agri - CDM | Urban | 1.6884 | 0.0913 | two.sided | z | None | Dunn |
| PFOS | Agri - CDM | Wood | 3.0439 | 0.0023 | two.sided | z | None | Dunn |
| PFOS | Agri - VAC | Urban | 0.6467 | 0.5178 | two.sided | z | None | Dunn |
| PFOS | Agri - VAC | Wood | 1.9073 | 0.0565 | two.sided | z | None | Dunn |
| PFOS | Urban | Wood | 1.5987 | 0.1099 | two.sided | z | None | Dunn |
| ∑PFCAs | Agri - CDM | Agri - VAC | 1.1249 | 0.2606 | two.sided | z | None | Dunn |
| ∑PFCAs | Agri - CDM | Urban | 1.7378 | 0.0822 | two.sided | z | None | Dunn |
| ∑PFCAs | Agri - CDM | Wood | 1.5219 | 0.1280 | two.sided | z | None | Dunn |
| ∑PFCAs | Agri - VAC | Urban | 0.2841 | 0.7764 | two.sided | z | None | Dunn |
| ∑PFCAs | Agri - VAC | Wood | 0.2194 | 0.8263 | two.sided | z | None | Dunn |
| ∑PFCAs | Urban | Wood | 0.0551 | 0.9560 | two.sided | z | None | Dunn |
| PFOA | Agri - CDM | Agri - VAC | 0.2756 | 0.7828 | two.sided | z | None | Dunn |
| PFOA | Agri - CDM | Urban | 0.6589 | 0.5100 | two.sided | z | None | Dunn |
| PFOA | Agri - CDM | Wood | 1.2757 | 0.2020 | two.sided | z | None | Dunn |
| PFOA | Agri - VAC | Urban | 0.8401 | 0.4009 | two.sided | z | None | Dunn |
| PFOA | Agri - VAC | Wood | 1.3672 | 0.1716 | two.sided | z | None | Dunn |
| PFOA | Urban | Wood | 0.7166 | 0.4736 | two.sided | z | None | Dunn |
| PFNA | Agri - CDM | Agri - VAC | 2.3004 | 0.0214 | two.sided | z | None | Dunn |
| PFNA | Agri - CDM | Urban | 0.8944 | 0.3711 | two.sided | z | None | Dunn |
| PFNA | Agri - CDM | Wood | 1.6816 | 0.0927 | two.sided | z | None | Dunn |
| PFNA | Agri - VAC | Urban | 1.6866 | 0.0917 | two.sided | z | None | Dunn |
| PFNA | Agri - VAC | Wood | 0.6957 | 0.4866 | two.sided | z | None | Dunn |
| PFNA | Urban | Wood | 0.9804 | 0.3269 | two.sided | z | None | Dunn |
| PFDA | Agri - CDM | Agri - VAC | 0.3484 | 0.7275 | two.sided | z | None | Dunn |
| PFDA | Agri - CDM | Urban | 0.3509 | 0.7256 | two.sided | z | None | Dunn |
| PFDA | Agri - CDM | Wood | 0.1525 | 0.8788 | two.sided | z | None | Dunn |
| PFDA | Agri - VAC | Urban | 0.0691 | 0.9449 | two.sided | z | None | Dunn |
| PFDA | Agri - VAC | Wood | 0.1924 | 0.8474 | two.sided | z | None | Dunn |
| PFDA | Urban | Wood | 0.1570 | 0.8753 | two.sided | z | None | Dunn |
| PFUnDA | Agri - CDM | Agri - VAC | 0.1193 | 0.9050 | two.sided | z | None | Dunn |
| PFUnDA | Agri - CDM | Urban | 0.1977 | 0.8433 | two.sided | z | None | Dunn |
| PFUnDA | Agri - CDM | Wood | 1.5259 | 0.1270 | two.sided | z | None | Dunn |
| PFUnDA | Agri - VAC | Urban | 0.2983 | 0.7654 | two.sided | z | None | Dunn |
| PFUnDA | Agri - VAC | Wood | 1.4922 | 0.1357 | two.sided | z | None | Dunn |
| PFUnDA | Urban | Wood | 1.3902 | 0.1645 | two.sided | z | None | Dunn |
| PFDoDA | Agri - CDM | Agri - VAC | 1.1536 | 0.2487 | two.sided | z | None | Dunn |
| PFDoDA | Agri - CDM | Urban | 2.9401 | 0.0033 | two.sided | z | None | Dunn |
| PFDoDA | Agri - CDM | Wood | 2.0818 | 0.0374 | two.sided | z | None | Dunn |
| PFDoDA | Agri - VAC | Urban | 1.2624 | 0.2068 | two.sided | z | None | Dunn |
| PFDoDA | Agri - VAC | Wood | 0.7393 | 0.4597 | two.sided | z | None | Dunn |
| PFDoDA | Urban | Wood | 0.4710 | 0.6377 | two.sided | z | None | Dunn |
| PFTrDA | Agri - CDM | Agri - VAC | 1.8468 | 0.0648 | two.sided | z | None | Dunn |
| PFTrDA | Agri - CDM | Urban | 1.1413 | 0.2537 | two.sided | z | None | Dunn |
| PFTrDA | Agri - CDM | Wood | 2.4623 | 0.0138 | two.sided | z | None | Dunn |
| PFTrDA | Agri - VAC | Urban | 1.1551 | 0.2481 | two.sided | z | None | Dunn |
| PFTrDA | Agri - VAC | Wood | 0.1343 | 0.8931 | two.sided | z | None | Dunn |
| PFTrDA | Urban | Wood | 1.7990 | 0.0720 | two.sided | z | None | Dunn |
| PFTeDA | Agri - CDM | Agri - VAC | 1.4105 | 0.1584 | two.sided | z | None | Dunn |
| PFTeDA | Agri - CDM | Urban | 1.1151 | 0.2648 | two.sided | z | None | Dunn |
| PFTeDA | Agri - CDM | Wood | 1.3124 | 0.1894 | two.sided | z | None | Dunn |
| PFTeDA | Agri - VAC | Urban | 2.2862 | 0.0222 | two.sided | z | None | Dunn |
| PFTeDA | Agri - VAC | Wood | 2.3792 | 0.0173 | two.sided | z | None | Dunn |
| PFTeDA | Urban | Wood | 0.3629 | 0.7167 | two.sided | z | None | Dunn |

Table S8. The Kaiser-Meyer-Olkin (KMO) measure of sampling adequacy (MSA) showed the usefulness of the Principal Component Analysis (PCA) for the PFAS analysis in ng/g ww.

|  | **MSA** | **%** |
| --- | --- | --- |
| Overall | 0.68 | - |
| PFOS | 0.42 | 0 |
| PFOA | 0.61 | 0 |
| PFNA | 0.68 | 8 |
| PFDA | 0.66 | 0 |
| PFUnDA | 0.78 | 15 |
| PFDoDA | 0.63 | 4 |
| PFTrDA | 0.80 | 31 |
| PFTeDA | 0.83 | 31 |

Table S9. Kruskal–Wallis one-way analysis of variance (p<0.05). In red those that shows significant differences in OCP concentrations (ng/g lw) among locations. In red are highlighted the statistically significant results.

| **id** | **parameter1** | **parameter2** | **statistic** | **df.error** | **p.value** | **method** | **effectsize** | **estimate** | **conf.level** | **conf.low** | **conf.high** | **conf.method** | **conf.iterations** | **n.obs** |
| --- | --- | --- | --- | --- | --- | --- | --- | --- | --- | --- | --- | --- | --- | --- |
| ∑OCPs | level | Area_type | 16.8857 | 3 | 0.0007 | Kruskal-Wallis rank sum test | Epsilon2 (rank) | 0.8887 | 0.95 | 0.8841 | 1 | percentile bootstrap | 100 | 20 |
| ∑DDTs | level | Area_type | 16.7905 | 3 | 0.0008 | Kruskal-Wallis rank sum test | Epsilon2 (rank) | 0.8837 | 0.95 | 0.8837 | 1 | percentile bootstrap | 100 | 20 |
| o,p'-DDE | level | Area_type | 2.1000 | 3 | 0.5519 | Kruskal-Wallis rank sum test | Epsilon2 (rank) | 0.1312 | 0.95 | 0.0733 | 1 | percentile bootstrap | 100 | 17 |
| p,p'-DDE | level | Area_type | 16.7905 | 3 | 0.0008 | Kruskal-Wallis rank sum test | Epsilon2 (rank) | 0.8837 | 0.95 | 0.8837 | 1 | percentile bootstrap | 100 | 20 |
| o,p'-DDD | level | Area_type | 0.3809 | 3 | 0.9442 | Kruskal-Wallis rank sum test | Epsilon2 (rank) | 0.0254 | 0.95 | 0.0218 | 1 | percentile bootstrap | 100 | 16 |
| p,p'-DDD | level | Area_type | 6.2472 | 3 | 0.1002 | Kruskal-Wallis rank sum test | Epsilon2 (rank) | 0.3288 | 0.95 | 0.1265 | 1 | percentile bootstrap | 100 | 20 |
| o,p'-DDT | level | Area_type | 6.6735 | 3 | 0.0831 | Kruskal-Wallis rank sum test | Epsilon2 (rank) | 0.3512 | 0.95 | 0.2439 | 1 | percentile bootstrap | 100 | 20 |
| p,p'-DDT | level | Area_type | 7.8129 | 3 | 0.0500 | Kruskal-Wallis rank sum test | Epsilon2 (rank) | 0.4112 | 0.95 | 0.2798 | 1 | percentile bootstrap | 100 | 20 |
| ∑endosulfan | level | Area_type | 1.4431 | 3 | 0.6955 | Kruskal-Wallis rank sum test | Epsilon2 (rank) | 0.0760 | 0.95 | 0.0308 | 1 | percentile bootstrap | 100 | 20 |
| α-endosulfan | level | Area_type | 1.4332 | 3 | 0.6978 | Kruskal-Wallis rank sum test | Epsilon2 (rank) | 0.0796 | 0.95 | 0.0258 | 1 | percentile bootstrap | 100 | 19 |
| ∑HCHs | level | Area_type | 2.7392 | 3 | 0.4336 | Kruskal-Wallis rank sum test | Epsilon2 (rank) | 0.1442 | 0.95 | 0.0668 | 1 | percentile bootstrap | 100 | 20 |
| α-HCH | level | Area_type | 0.7181 | 3 | 0.8689 | Kruskal-Wallis rank sum test | Epsilon2 (rank) | 0.0422 | 0.95 | 0.0219 | 1 | percentile bootstrap | 100 | 18 |
| β-HCH | level | Area_type | 5.4317 | 3 | 0.1428 | Kruskal-Wallis rank sum test | Epsilon2 (rank) | 0.2859 | 0.95 | 0.1428 | 1 | percentile bootstrap | 100 | 20 |
| γ-HCH | level | Area_type | 4.7088 | 3 | 0.1944 | Kruskal-Wallis rank sum test | Epsilon2 (rank) | 0.2478 | 0.95 | 0.1739 | 1 | percentile bootstrap | 100 | 20 |
| HCB | level | Area_type | 7.5901 | 3 | 0.0553 | Kruskal-Wallis rank sum test | Epsilon2 (rank) | 0.3995 | 0.95 | 0.2822 | 1 | percentile bootstrap | 100 | 20 |
| PeCB | level | Area_type | 2.7327 | 3 | 0.4347 | Kruskal-Wallis rank sum test | Epsilon2 (rank) | 0.1438 | 0.95 | 0.0653 | 1 | percentile bootstrap | 100 | 20 |

Table S10. Dunn’s Test for multiple comparisons (p<0.05). In red those that shows significant differences in OCP concentrations (ng/g lw) among locations. In red are highlighted the statistically significant results.

| **id** | **group1** | **group2** | **statistic** | **p.value** | **alternative** | **distribution** | **p.adjust.method** | **test** |
| --- | --- | --- | --- | --- | --- | --- | --- | --- |
| ∑OCPs | Agri - CDM | Agri - VAC | 3.0305 | 0.0024 | two.sided | z | None | Dunn |
| ∑OCPs | Agri - CDM | Urban | 1.7321 | 0.0833 | two.sided | z | None | Dunn |
| ∑OCPs | Agri - CDM | Wood | 3.6289 | 0.0003 | two.sided | z | None | Dunn |
| ∑OCPs | Agri - VAC | Urban | 1.8974 | 0.0578 | two.sided | z | None | Dunn |
| ∑OCPs | Agri - VAC | Wood | 0.4140 | 0.6788 | two.sided | z | None | Dunn |
| ∑OCPs | Urban | Wood | 2.1268 | 0.0334 | two.sided | z | None | Dunn |
| ∑DDTs | Agri - CDM | Agri - VAC | 2.9294 | 0.0034 | two.sided | z | None | Dunn |
| ∑DDTs | Agri - CDM | Urban | 1.7321 | 0.0833 | two.sided | z | None | Dunn |
| ∑DDTs | Agri - CDM | Wood | 3.6754 | 0.0002 | two.sided | z | None | Dunn |
| ∑DDTs | Agri - VAC | Urban | 1.7920 | 0.0731 | two.sided | z | None | Dunn |
| ∑DDTs | Agri - VAC | Wood | 0.2760 | 0.7825 | two.sided | z | None | Dunn |
| ∑DDTs | Urban | Wood | 2.1774 | 0.0295 | two.sided | z | None | Dunn |
| o,p'-DDE | Agri - CDM | Agri - VAC | 1.1598 | 0.2461 | two.sided | z | None | Dunn |
| o,p'-DDE | Agri - CDM | Urban | 0.3985 | 0.6902 | two.sided | z | None | Dunn |
| o,p'-DDE | Agri - CDM | Wood | 0.1308 | 0.8959 | two.sided | z | None | Dunn |
| o,p'-DDE | Agri - VAC | Urban | 1.4292 | 0.1530 | two.sided | z | None | Dunn |
| o,p'-DDE | Agri - VAC | Wood | 1.0914 | 0.2751 | two.sided | z | None | Dunn |
| o,p'-DDE | Urban | Wood | 0.5369 | 0.5914 | two.sided | z | None | Dunn |
| p,p'-DDE | Agri - CDM | Agri - VAC | 2.9294 | 0.0034 | two.sided | z | None | Dunn |
| p,p'-DDE | Agri - CDM | Urban | 1.7321 | 0.0833 | two.sided | z | None | Dunn |
| p,p'-DDE | Agri - CDM | Wood | 3.6754 | 0.0002 | two.sided | z | None | Dunn |
| p,p'-DDE | Agri - VAC | Urban | 1.7920 | 0.0731 | two.sided | z | None | Dunn |
| p,p'-DDE | Agri - VAC | Wood | 0.2760 | 0.7825 | two.sided | z | None | Dunn |
| p,p'-DDE | Urban | Wood | 2.1774 | 0.0295 | two.sided | z | None | Dunn |
| p,p'-DDD | Agri - CDM | Agri - VAC | 0.3515 | 0.7252 | two.sided | z | None | Dunn |
| p,p'-DDD | Agri - CDM | Urban | 0.5944 | 0.5522 | two.sided | z | None | Dunn |
| p,p'-DDD | Agri - CDM | Wood | 0.3122 | 0.7549 | two.sided | z | None | Dunn |
| p,p'-DDD | Agri - VAC | Urban | 0.1534 | 0.8781 | two.sided | z | None | Dunn |
| p,p'-DDD | Agri - VAC | Wood | 0.1286 | 0.8977 | two.sided | z | None | Dunn |
| p,p'-DDD | Urban | Wood | 0.3466 | 0.7289 | two.sided | z | None | Dunn |
| p,p'-DDD | Agri - CDM | Agri - VAC | 0.7475 | 0.4548 | two.sided | z | None | Dunn |
| p,p'-DDD | Agri - CDM | Urban | 1.6248 | 0.1042 | two.sided | z | None | Dunn |
| p,p'-DDD | Agri - CDM | Wood | 0.5955 | 0.5515 | two.sided | z | None | Dunn |
| p,p'-DDD | Agri - VAC | Urban | 0.4066 | 0.6843 | two.sided | z | None | Dunn |
| p,p'-DDD | Agri - VAC | Wood | 1.2076 | 0.2272 | two.sided | z | None | Dunn |
| p,p'-DDD | Urban | Wood | 2.3582 | 0.0184 | two.sided | z | None | Dunn |
| o,p'-DDT | Agri - CDM | Agri - VAC | 1.0102 | 0.3124 | two.sided | z | None | Dunn |
| o,p'-DDT | Agri - CDM | Urban | 1.6496 | 0.0990 | two.sided | z | None | Dunn |
| o,p'-DDT | Agri - CDM | Wood | 0.0000 | 1.0000 | two.sided | z | None | Dunn |
| o,p'-DDT | Agri - VAC | Urban | 2.2588 | 0.0239 | two.sided | z | None | Dunn |
| o,p'-DDT | Agri - VAC | Wood | 1.0351 | 0.3006 | two.sided | z | None | Dunn |
| o,p'-DDT | Urban | Wood | 1.7361 | 0.0825 | two.sided | z | None | Dunn |
| p,p'-DDT | Agri - CDM | Agri - VAC | 0.5051 | 0.6135 | two.sided | z | None | Dunn |
| p,p'-DDT | Agri - CDM | Urban | 1.8558 | 0.0635 | two.sided | z | None | Dunn |
| p,p'-DDT | Agri - CDM | Wood | 0.4652 | 0.6418 | two.sided | z | None | Dunn |
| p,p'-DDT | Agri - VAC | Urban | 1.8823 | 0.0598 | two.sided | z | None | Dunn |
| p,p'-DDT | Agri - VAC | Wood | 0.1725 | 0.8630 | two.sided | z | None | Dunn |
| p,p'-DDT | Urban | Wood | 2.4595 | 0.0139 | two.sided | z | None | Dunn |
| ∑endosulfan | Agri - CDM | Agri - VAC | 0.6465 | 0.5180 | two.sided | z | None | Dunn |
| ∑endosulfan | Agri - CDM | Urban | 0.2722 | 0.7855 | two.sided | z | None | Dunn |
| ∑endosulfan | Agri - CDM | Wood | 0.6606 | 0.5088 | two.sided | z | None | Dunn |
| ∑endosulfan | Agri - VAC | Urban | 0.8734 | 0.3825 | two.sided | z | None | Dunn |
| ∑endosulfan | Agri - VAC | Wood | 0.1725 | 0.8630 | two.sided | z | None | Dunn |
| ∑endosulfan | Urban | Wood | 1.0055 | 0.3147 | two.sided | z | None | Dunn |
| α-endosulfan | Agri - CDM | Agri - VAC | 0.4036 | 0.6865 | two.sided | z | None | Dunn |
| α-endosulfan | Agri - CDM | Urban | 1.1619 | 0.2453 | two.sided | z | None | Dunn |
| α-endosulfan | Agri - CDM | Wood | 0.8429 | 0.3993 | two.sided | z | None | Dunn |
| α-endosulfan | Agri - VAC | Urban | 0.4274 | 0.6691 | two.sided | z | None | Dunn |
| α-endosulfan | Agri - VAC | Wood | 0.2336 | 0.8153 | two.sided | z | None | Dunn |
| α-endosulfan | Urban | Wood | 0.2515 | 0.8015 | two.sided | z | None | Dunn |
| β-emdosulfan | Agri - CDM | Urban | 1.3522 | 0.1763 | two.sided | z | None | Dunn |
| β-emdosulfan | Agri - CDM | Wood | 1.6366 | 0.1017 | two.sided | z | None | Dunn |
| β-emdosulfan | Urban | Wood | 0.7606 | 0.4469 | two.sided | z | None | Dunn |
| ∑HCHs | Agri - CDM | Agri - VAC | 0.1818 | 0.8557 | two.sided | z | None | Dunn |
| ∑HCHs | Agri - CDM | Urban | 1.4104 | 0.1584 | two.sided | z | None | Dunn |
| ∑HCHs | Agri - CDM | Wood | 0.4466 | 0.6551 | two.sided | z | None | Dunn |
| ∑HCHs | Agri - VAC | Urban | 1.2197 | 0.2226 | two.sided | z | None | Dunn |
| ∑HCHs | Agri - VAC | Wood | 0.5175 | 0.6048 | two.sided | z | None | Dunn |
| ∑HCHs | Urban | Wood | 0.9983 | 0.3181 | two.sided | z | None | Dunn |
| α-HCH | Agri - CDM | Agri - VAC | 0.8060 | 0.4202 | two.sided | z | None | Dunn |
| α-HCH | Agri - CDM | Urban | 0.4739 | 0.6356 | two.sided | z | None | Dunn |
| α-HCH | Agri - CDM | Wood | 0.2372 | 0.8125 | two.sided | z | None | Dunn |
| α-HCH | Agri - VAC | Urban | 0.4478 | 0.6543 | two.sided | z | None | Dunn |
| α-HCH | Agri - VAC | Wood | 0.6500 | 0.5157 | two.sided | z | None | Dunn |
| α-HCH | Urban | Wood | 0.2578 | 0.7966 | two.sided | z | None | Dunn |
| β-HCH | Agri - CDM | Agri - VAC | 1.7375 | 0.0823 | two.sided | z | None | Dunn |
| β-HCH | Agri - CDM | Urban | 1.6578 | 0.0974 | two.sided | z | None | Dunn |
| β-HCH | Agri - CDM | Wood | 0.2140 | 0.8305 | two.sided | z | None | Dunn |
| β-HCH | Agri - VAC | Urban | 0.6023 | 0.5469 | two.sided | z | None | Dunn |
| β-HCH | Agri - VAC | Wood | 1.6217 | 0.1049 | two.sided | z | None | Dunn |
| β-HCH | Urban | Wood | 1.5119 | 0.1306 | two.sided | z | None | Dunn |
| γ-HCH | Agri - CDM | Agri - VAC | 1.5152 | 0.1297 | two.sided | z | None | Dunn |
| γ-HCH | Agri - CDM | Urban | 0.3299 | 0.7415 | two.sided | z | None | Dunn |
| γ-HCH | Agri - CDM | Wood | 0.7909 | 0.4290 | two.sided | z | None | Dunn |
| γ-HCH | Agri - VAC | Urban | 1.8221 | 0.0684 | two.sided | z | None | Dunn |
| γ-HCH | Agri - VAC | Wood | 2.1392 | 0.0324 | two.sided | z | None | Dunn |
| γ-HCH | Urban | Wood | 0.5136 | 0.6075 | two.sided | z | None | Dunn |
| HCB | Agri - CDM | Agri - VAC | 2.0607 | 0.0393 | two.sided | z | None | Dunn |
| HCB | Agri - CDM | Urban | 1.0475 | 0.2949 | two.sided | z | None | Dunn |
| HCB | Agri - CDM | Wood | 0.5490 | 0.5830 | two.sided | z | None | Dunn |
| HCB | Agri - VAC | Urban | 1.3854 | 0.1659 | two.sided | z | None | Dunn |
| HCB | Agri - VAC | Wood | 2.5187 | 0.0118 | two.sided | z | None | Dunn |
| HCB | Urban | Wood | 1.7000 | 0.0891 | two.sided | z | None | Dunn |
| PeCB | Agri - CDM | Agri - VAC | 0.3030 | 0.7619 | two.sided | z | None | Dunn |
| PeCB | Agri - CDM | Urban | 1.1959 | 0.2317 | two.sided | z | None | Dunn |
| PeCB | Agri - CDM | Wood | 1.1166 | 0.2642 | two.sided | z | None | Dunn |
| PeCB | Agri - VAC | Urban | 1.1896 | 0.2342 | two.sided | z | None | Dunn |
| PeCB | Agri - VAC | Wood | 1.1386 | 0.2549 | two.sided | z | None | Dunn |
| PeCB | Urban | Wood | 0.0434 | 0.9654 | two.sided | z | None | Dunn |

Table S11. The Kaiser-Meyer-Olkin (KMO) measure of sampling adequacy (MSA) showed the usefulness of the Principal Component Analysis (PCA) for the OCPs analysis in ng/g lw.

|  | **MSA** | **%** |
| --- | --- | --- |
| Overall | 0.51 | - |
| o,p'-DDD | 0.56 | 20 |
| o,p'-DDE | 0.61 | 15 |
| o,p'-DDT | 0.32 | 0 |
| p,p'-DDD | 0.53 | 0 |
| p,p'-DDE | 0.53 | 0 |
| p,p'-DDT | 0.53 | 0 |
| α-endosulfan | 0.68 | 1 |
| β-endosulfan | 0.71 | 65 |
| α-HCH | 0.6 | 10 |
| β-HCH | 0.14 | 0 |
| γ-HCH | 0.27 | 0 |
| PeCB | 0.58 | 0 |
| HCB | 0.3 | 0 |

Table S12. The Kaiser-Meyer-Olkin (KMO) measure of sampling adequacy (MSA) showed the usefulness of the Principal Component Analysis (PCA) for the OCPs analysis in ng/g ww.

|  | | **MSA** | **%** | |
| --- | --- | --- | --- | --- |
| Overall | 0.81 | | | - |
| op'-DDD | | 0.73 | 20 | |
| op'-DDE | | 0.74 | 15 | |
| op'-DDT | | 0.80 | 0 | |
| pp'-DDD | | 0.74 | 0 | |
| pp'-DDE | | 0.84 | 0 | |
| pp'-DDT | | 0.77 | 0 | |
| α-endosulfan | | 0.79 | 1 | |
| β-endosulfan | | 0.87 | 65 | |
| α-HCH | | 0.89 | 10 | |
| β-HCH | | 0.88 | 0 | |
| γ-HCH | | 0.78 | 0 | |
| PeCB | | 0.90 | 0 | |
| HCB | | 0.79 | 0 | |

Table S13. Levels of PFAS (ng/g ww) reported in previous studies compared to the present study.

| **Location** | **Type** | **Year** | **Statistic** | **PFOA** | **PFNA** | **PFDA** | **PFUnDA** | **PFDoDA** | **PFTrDA** | **PFTeDA** | **PFHxS** | **PFOS** | **PFDS** | **Author** |
| --- | --- | --- | --- | --- | --- | --- | --- | --- | --- | --- | --- | --- | --- | --- |
| Italy | Agricultural | 2021-2022 | Median | 0.30 | 0.49 | 0.82 | 0.29 | 0.69 | 0.63 | 0.16 |  | 5.31 | 0.032 | Present study |
|  | Overall | 2021-2022 | Median | 0.27 | 0.35 | 0.82 | 0.40 | 0.98 | 0.65 | 0.33 |  | 4.75 | 0.04 |  |
|  | Urban | 2021-2022 | Median | 0.24 | 0.28 | 0.87 | 0.32 | 1.75 | 0.46 | 0.81 |  | 3.65 | 0.04 |  |
|  | Woodland | 2021-2022 | Median | 0.22 | 0.42 | 0.74 | 0.53 | 1.13 | 1.02 | 0.50 |  | 2.23 |  |  |
| Belgium | Fluorochemcial Plant | 2011 | Median | 19.8 |  | 12 |  | 13.7 | 5.6 |  | 99.3 | 10380 | 47.7 | (Groffen et al., 2017) |
|  | Vlietbos | 2011 | Median | 0.9 |  |  |  | 1 |  |  |  | 125 |  |  |
|  | Rot | 2011 | Median | 0.8 |  |  |  |  |  |  |  | 107.1 |  |  |
|  | Tessenderlo | 2011 | Median | 0.3 |  |  |  | 0.6 | 0.5 |  |  | 9.4 |  |  |
|  | Fluorochemcial Plant | 2015 | Median | 18 | 7.7 | 130 |  | 18 | 14 | 1.3 |  | 34251 | 82 | (Groffen et al., 2019) |
|  | Ros | 2015 | Median | 1.4 | 1.4 | 1.4 |  | 2.9 | 6.6 | 1.2 |  | 454 |  |  |
|  | Vlietbos | 2015 | Median | 1.3 | 1.4 |  |  |  | 4.1 |  |  | 416 |  |  |
|  | Burchtse Weel | 2015 | Median | 1.5 | 1.2 |  |  | 1.6 | 2.5 |  |  | 87 |  |  |
|  | Fort | 2015 | Median | 1.2 | 1.3 | 1.9 |  | 1.6 | 2.5 | 0.7 |  | 30 |  |  |
|  | Fluorochemcial Plant | 2015 | Mean | 18.2 |  |  |  | 57.5 |  |  |  | 80231 |  | (Lopez-Antia et al., 2019) |
|  | Vlietbos | 2015 | Mean | 1.3 |  |  |  |  |  |  |  | 890 |  |  |
|  | Rot | 2015 | Mean | 1.2 |  |  |  | 1.7 |  |  |  | 351 |  |  |
|  | Burchtse Weel | 2015 | Mean | 1.1 |  |  |  |  |  |  |  | 80 |  |  |
|  | Fort | 2015 | Mean | 1 |  |  |  | 2 |  |  |  | 29.2 |  |  |

Table S14. Median levels of OCPs (ng/g lw) reported in previous studies compared to the present study.

| **Location** | **Year** | **PeCB** | **HCB** | **α-HCH** | **γ-HCH** | **β-HCH** | **∑HCHs** | **α-endo.** | **β-endo.** | **∑endo.** | **o,p'-DDE** | **pp'-DDE** | **o,p'-DDD** | **o,p'-DDT** | **pp'-DDD** | **pp'-DDT** | **∑DDTs** | **∑OCPs** | **Author** |
| --- | --- | --- | --- | --- | --- | --- | --- | --- | --- | --- | --- | --- | --- | --- | --- | --- | --- | --- | --- |
| Italy | 2021-2022 | 0.79 | 14.0 | 0.788 | 1.012 | 1.25 | 4.13 | 0.29 | 0.321 | 0.290 | 0.228 | 3140 | 0.113 | 0.382 | 2.91 | 10.5 | 3160 | 3180 | Present study |
|  | 2021-2022 | 0.952 | 12.4 | 0.826 | 0.732 | 0.900 | 2.25 | 0.201 | 0.188 | 0.342 | 0.167 | 1796 | 0.119 | 1.19 | 3.23 | 32.0 | 1830 | 1850 |  |
|  | 2021-2022 | 1.08 | 14.1 | 0.807 | 0.868 | 1.12 | 2.86 | 0.247 | 0.188 | 0.318 | LOD | 1675 | 0.116 | 0.616 | 2.96 | 17.7 | 1710 | 1730 |  |
|  | 2021-2022 | 1.14 | 20.4 | 0.762 | 0.605 | 1.20 | 2.84 | 0.186 | 0.182 | 0.292 | 0.233 | 628 | 0.11 | 0.557 | 1.6 | 12.5 | 642 | 670 |  |
| Belgium | 2005 |  | 19.9 |  |  |  |  |  |  |  |  | 504 |  |  |  |  |  |  | (Dauwe et al., 2006) |
|  | 2005 |  | 22.2 |  |  |  |  |  |  |  |  | 486 |  |  |  |  |  |  |  |
| Belgium | 2006 |  | 24.8 |  |  | 8.00 |  |  |  |  |  | 661 |  |  | 4.80 | 19.1 |  | 1020 | (Van den Steen et al., 2009) |
| Czech R. | 2006 |  | 60.5 |  |  | 1.18 |  |  |  |  |  | 2420 |  |  | 10.4 | 90.5 |  | 2560 |  |
| Germany | 2006 |  | 26.8 | 0.400 | 0.350 | 8.10 |  |  |  |  |  | 344 |  |  |  | 14.2 |  | 433 |  |
| Estonia | 2006 |  | 41.0 | 0.300 |  | 7.90 |  |  |  |  |  | 420 |  | 3.00 | 10.8 | 5.40 |  | 486 |  |
| France | 2006 |  | 15.3 | 0.500 | 9.80 | 10.4 |  |  |  |  |  | 439 |  |  |  | 4.00 |  | 480.4 |  |
| Finland | 2006 |  | 16.6 | 0.300 | 4.50 | 4.10 |  |  |  |  |  | 117 |  |  |  | 3.60 |  | 157 |  |
| Hungary | 2006 |  | 15.2 | 0.300 | 5.00 | 7.20 |  |  |  |  |  | 1130 |  |  |  | 18.0 |  | 1190 |  |
| Italy | 2006 |  | 13.7 | 0.300 | 5.10 | 2.40 |  |  |  |  |  | 261 |  | 11.2 |  | 5.40 |  | 357 |  |
| Nederland | 2006 |  | 24.7 | 0.300 |  | 5.90 |  |  |  |  |  | 366 |  |  |  | 8.70 |  | 424 |  |
| Norway | 2006 |  | 18.2 | 0.400 | 5.00 | 1.60 |  |  |  |  |  | 188 |  |  |  | 5.20 |  | 238 |  |
| Poland | 2006 |  | 34.0 | 0.800 | 4.40 | 10.3 |  |  |  |  |  | 2280 |  | 4.70 | 7.40 | 48.7 |  | 2430 |  |
| Portugal | 2006 |  | 2.80 |  |  | 2.40 |  |  |  |  |  | 295 |  | 0.900 | 3.60 | 2.00 |  | 315 |  |
| Spain | 2006 |  | 6.90 | 0.300 | 3.30 | 7.60 |  |  |  |  |  | 535 |  | 7.30 | 193 | 5.60 |  | 602 |  |
| Sweden | 2006 |  | 29.9 | 0.300 | 7.00 | 5.60 |  |  |  |  |  | 818 |  |  | 10.6 | 15.7 |  | 888 |  |

Bibliography

Dauwe, T., Jaspers, V.L.B., Covaci, A., Eens, M., 2006. Accumulation of Organochlorines and Brominated Flame Retardants in the Eggs and Nestlings of Great Tits, Parus major. Environ Sci Technol 40, 5297–5303. https://doi.org/10.1021/es060747a

Groffen, T., Lasters, R., Lopez-Antia, A., Prinsen, E., Bervoets, L., Eens, M., 2019. Limited reproductive impairment in a passerine bird species exposed along a perfluoroalkyl acid (PFAA) pollution gradient. Science of The Total Environment 652, 718–728. https://doi.org/https://doi.org/10.1016/j.scitotenv.2018.10.273

Groffen, T., Lopez-Antia, A., D’Hollander, W., Prinsen, E., Eens, M., Bervoets, L., 2017. Perfluoroalkylated acids in the eggs of great tits (*Parus major*) near a fluorochemical plant in Flanders, Belgium. Environmental Pollution 228, 140–148. https://doi.org/10.1016/j.envpol.2017.05.007

Lopez-Antia, A., Groffen, T., Lasters, R., AbdElgawad, H., Sun, J., Asard, H., Bervoets, L., Eens, M., 2019. Perfluoroalkyl Acids (PFAAs) Concentrations and Oxidative Status in Two Generations of Great Tits Inhabiting a Contamination Hotspot. Environ Sci Technol 53, 1617–1626. https://doi.org/10.1021/acs.est.8b05235

Mazzoni, M., Polesello, S., Rusconi, M., Valsecchi, S., 2016. Liquid chromatography mass spectrometry determination of perfluoroalkyl acids in environmental solid extracts after phospholipid removal and on-line turbulent flow chromatography purification. J Chromatogr A 1453, 62–70. https://doi.org/10.1016/j.chroma.2016.05.047

Van den Steen, E., Pinxten, R., Jaspers, V.L.B., Covaci, A., Barba, E., Carere, C., Cichoń, M., Dubiec, A., Eeva, T., Heeb, P., Kempenaers, B., Lifjeld, J.T., Lubjuhn, T., Mänd, R., Massa, B., Nilsson, J.-Å., Norte, A.C., Orell, M., Podzemny, P., Sanz, J.J., Senar, J.C., Soler, J.J., Sorace, A., Török, J., Visser, M.E., Winkel, W., Eens, M., 2009. Brominated flame retardants and organochlorines in the European environment using great tit eggs as a biomonitoring tool. Environ Int 35, 310–317. https://doi.org/10.1016/j.envint.2008.08.002
